# N^6^-methyladenosine RNA base modification regulates NKG2D-dependent and cytotoxic genes expression in natural killer cells

**DOI:** 10.1186/s12920-025-02147-y

**Published:** 2025-05-19

**Authors:** Raghda A. Elsabbagh, Ghada Abdelhady, Doris Urlaub, Mina Sandusky, Ola Khorshid, Mohamed Z. Gad, Khaled Abou-Aisha, Carsten Watzl, Mona Rady

**Affiliations:** 1 Biochemistry Department, Faculty of Pharmacy and Biotechnology, the German University in Cairo, Cairo, Egypt; 2 Microbiology, Immunology and Biotechnology Department, Faculty of Pharmacy and Biotechnology, the German University in Cairo, Cairo, Egypt; 3 Leibniz Research Centre for Working Environment and Human Factors (IfADo), TU Dortmund, Dortmund, Germany; 4 Medical Oncology Department, National Cancer Institute, Cairo University, Cairo, Egypt; 5 Faculty of Biotechnology, German International University, New Administrative Capital, Egypt

**Keywords:** Natural Killer Cells, N^6^ methyl adenosine, m^6^A erasers, FTO, ALKBH5, Breast Cancer, NKG2D, Related pathway, Lytic, Dependent pathway, Lytic, Independent pathway

## Abstract

**Background:**

Breast cancer (BC) is the most commonly diagnosed cancer in women. N^6^-methyladenosine (m^6^A) is the most prevalent internal modification in mammalian mRNAs and plays a crucial role in various biological processes. However, its function in Natural killer (NK) cells in BC remains unclear. NK cells are essential for cancer immunosurveillance. This study aims to assess m^6^A levels in transcripts involved in the NKG2D cytotoxicity signaling pathway in NK cells of BC patients compared to controls and find out its impact on mRNA levels. Additionally, it evaluates how deliberately altering m^6^A levels in NK cells affects mRNA and protein expression of NKG2D pathway genes and NK cell functionality.

**Methods:**

m^6^A methylation in transcripts of NKG2D-pathway-related genes in BC patients and controls was determined using methylated RNA immunoprecipitation-reverse transcription-PCR (MERIP-RT-PCR). To deliberately alter m^6^A levels in primary cultured human NK cells, the m^6^A demethylases, FTO and ALKBH5, were knocked out using the CRISPR-CAS9 system, and FTO was inhibited using Meclofenamic acid (MA). The impact of m^6^A alteration on corresponding mRNA and protein levels was assessed using RT-qPCR and Western blot analysis or flow cytometry, respectively. Additionally, NK cell functionality was evaluated through degranulation and ^51^Cr release cytotoxicity assays.

**Results:**

Transcripts of NKG2D, an activating receptor that detects stressed non-self tumour cells, had significantly higher m^6^A levels in the 3′ untranslated region (3’UTR) accompanied by a marked reduction in their corresponding mRNA levels in BC patients compared to controls. Conversely, transcripts of ERK2 and PRF1 exhibited significantly lower m^6^A levels escorted with higher mRNA expression in BC patients relative to controls. The mRNA levels of PI3K, PAK1 and GZMH were also significantly elevated in BC patients. Furthermore, artificially increasing transcripts’ m^6^A levels via MA in cultured primary NK cells reduced mRNA levels of NKG2D pathway genes and death receptor ligands but did not affect protein expression or NK cell functionality.

**Conclusion:**

Transcripts with higher m^6^A levels in the 3’UTR region were less abundant, and vice versa. However, changes in mRNA levels of the target genes didn’t impact their corresponding protein levels or NK cell functionality.

**Supplementary Information:**

The online version contains supplementary material available at 10.1186/s12920-025-02147-y.

## Background

Natural Killer (NK) cells play a crucial role in cancer immunosurveillance, serving as the first line of defense against transformed and virally infected cells. Their activation is tightly regulated by a balance of activating and inhibitory receptors on the cell surface [1]. Among these, the Natural Killer Group 2D (NKG2D) receptor, also known as Killer Cell Lectin Like Receptor K1 (KLRK1), is one of the most important activating receptors expressed on the surface of all NK cells.

NKG2D detects stressed non-self tumour cells and consequently triggers a cytoplasmic signaling cascade that leads to a cytotoxic and cytokine-mediated response. This signaling is highly dependent on DNAX‐activation protein 10 (DAP10), which, upon activation, binds to the p85 subunit of phosphatidyl-inositol-3-kinase (PI3K). PI3K triggers a sequential activation of the VAV1-Rac-Pak-MEK-ERK pathway, ultimately leading to the elimination of target cells [2]. (Rac: GTP Binding Protein Rac1, PAK: p21- activated kinase 1, MEK: Mitogen-activated protein kinase kinase, ERK: Extracellular signal-regulated kinase).

Activated NK cells liquidate their targets through two major mechanisms, both requiring direct contact between NK cells and target cells. The first mechanism involves the exocytosis of lytic granules containing the pore-forming protein, perforin (PRF1), and a family of serine proteases, granzymes (GZMs). Perforin forms pores in the plasma membrane of the target cell, allowing granzymes to enter and trigger apoptosis through both caspase-dependent and caspase-independent pathways [1, 3, 4]. The lysosomal-associated membrane protein-1 (LAMP1/CD107a) is externalized during this degranulation process and thereby lines the NK cell plasma membrane at the immunologic synapse, serving as a degranulation marker for active NK cells [5].

**Fig. 1:**
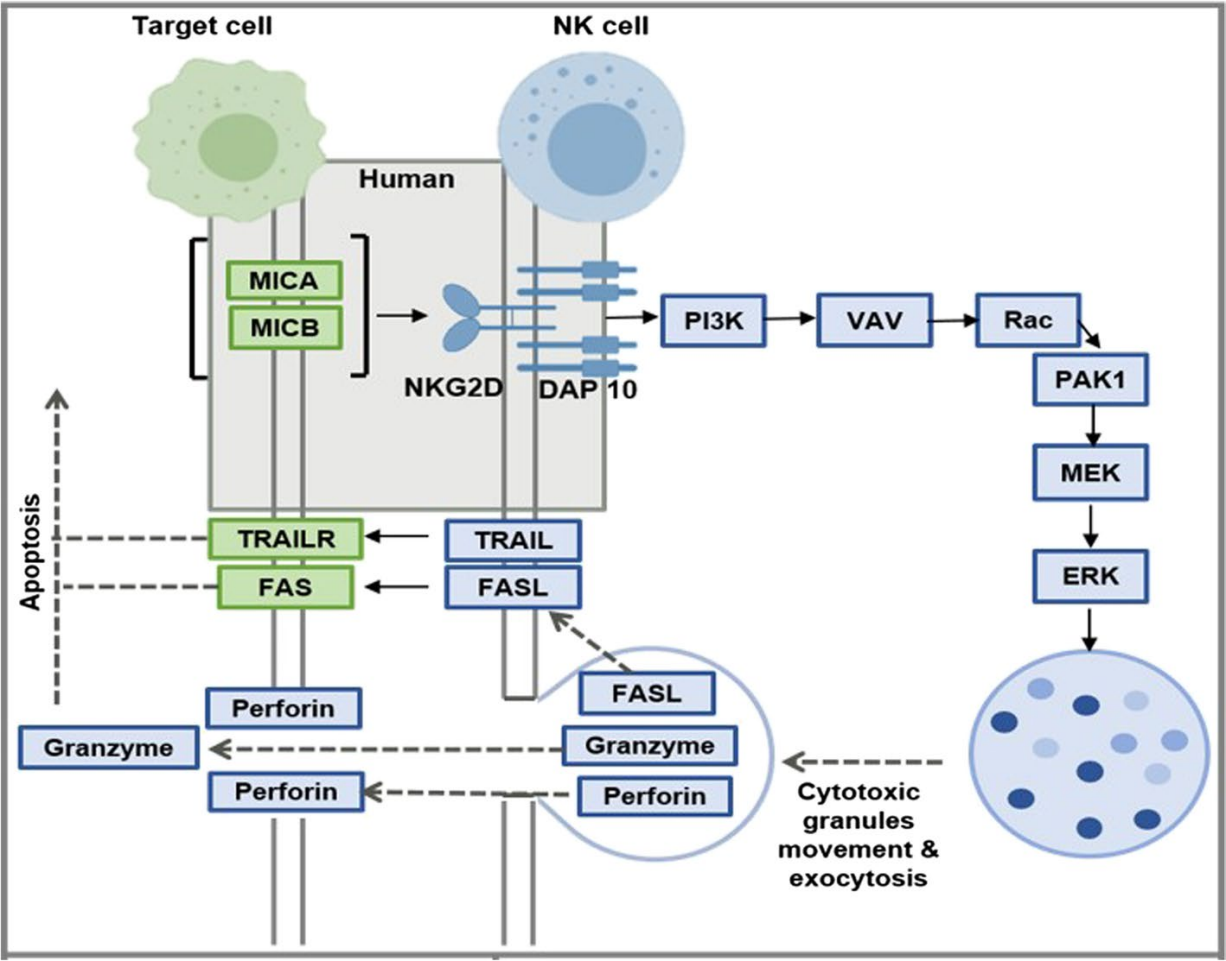
Natural killer cell-mediated cytotoxicity pathway

The second mechanism involves NK cells presenting Fas Ligand (FasL/CD95L) or TNF-related apoptosis-inducing ligand (TRAIL) on their surface. These ligands bind to their corresponding death receptors (CD95/Fas and TRAIL receptors) on the target cell, leading to classical caspase-dependent apoptosis [3, 4, 6, 7] **(**Fig. 1**)**.

Despite advancements in early diagnosis and treatment, breast cancer (BC) remains the most commonly diagnosed cancer worldwide, affecting 2.1 million women annually, according to the WHO [8] and ranking as the leading cause of cancer-related deaths among women worldwide [9]. BC is highly heterogeneous at the genetic, epigenetic, and phenotypic levels, making treatment strategies complex [10].

Cancer immunotherapy offers a promising approach by leveraging the immune system to eliminate tumors. It relies on the concept of immunosurveillance, where immune cells detect and destroy malignant cells during their early transformation [11]. Compared to chemotherapy and radiotherapy, immunotherapy is seen as a more targeted treatment with fewer adverse effects, particularly for metastatic breast cancer [12].

Studies have shown that patients with breast [13, 14], ovarian, lung [15], and cervical cancer exhibit reduced NK cell cytotoxicity, which is often associated with decreased NKG2D expression [16, 17]. Higher expression of NK cell activating markers has been linked to increased survival rates in BC patients, whereas NK cell dysfunction correlates with tumor progression [18].

It was shown that cancer patients' NK cells show reduced cytotoxicity compared to those of healthy donors. This has been attributed to the downregulation of activating markers, such as NKG2D and natural cytotoxicity receptors, at the protein level [19]. However, various studies have also demonstrated that mRNA expression does not always directly correlate with protein levels, suggesting that post-transcriptional modifications could be influencing NK cell function. These findings highlight the potential role of epi-transcriptomic regulation in NK cell-mediated cytotoxicity and, hence, a potential pathway for immunomodulation [20, 21].

The N^6^-methyladenosine (m^6^A) is the most abundant post-transcriptional modification in eukaryotic RNA transcripts [21]. It refers to the post-transcriptional methylation of the adenosine base at the nitrogen-6 position [22]. N^6^-methyladenosine bases are frequently enriched in the vicinity of stop codons, at the 3′ untranslated regions (3′ UTRs), in consensus sequences within long exons and at transcription start sites (TSSs) [23]. Adenosine methylation and demethylation is dynamic and reversible and both processes are accomplished by the orchestrated action of highly conserved methyl transferases (writers), such as Methyltransferase-like 3 (METTL3) and Methyltransferase-like 14 (METTL14), and demethylases (erasers), such as Fat mass and obesity-associated protein (FTO) and alkylated DNA repair protein AlkB homolog 5 (ALKBH5). Together, the writers and erasers shape the cellular ‘epitranscriptome’ [24]. The methyl code is decrypted by a group of m^6^A readers/effectors (YTH domain family proteins), which, in turn, govern the fate of the m^6^A-modified transcripts, thereby dictating their stability, transport, degradation, and translation [25, 26].

Recent studies have shown that the degree and pattern of N^6^-methyladenosine modifications can influence various aspects of mRNA processing, including splicing, storage, transport, stability, translation, and decay [25, 27]. It has been reported that the N^6^-methyladenosine modification plays a crucial regulatory role in multiple cellular processes and development, such as circadian rhythm maintenance, cell differentiation, reprogramming, state transitions and stress responses, thus shaping cell function and identity [24].

Abnormalities in N^6^-methyladenosine modifications have been linked to various diseases, including cancer [10, 28]. While the role of N^6^-methyladenosine has been extensively studied in different cancer cell types [10, 28–32] and some immune cells, such as T cells [33–35]; there is still limited research on its impact in NK cells [36–38].

Therefore, this study aims to investigate the N^6^-methyladenosine levels in some key transcripts within the NKG2D receptor pathway, e.g. (NKG2D, PIK3, VAV1, Pak1, ERK2), the lytic-dependent pathway (PRF1, GZMH), and the lytic-independent pathway/ligands of the death receptors (FASL and TRAIL) in NK cells from BC patients compared to healthy control subjects. We also explored how N^6^-methyladenosine modifications affect mRNA levels in these target genes in BC patients’ NK cells compared to controls. Finally, we examined whether deliberate alterations in N^6^-methyladenosine modification levels in primary cultured human NK cells influence corresponding mRNA levels, protein levels, and overall NK cell-mediated cytotoxicity.

## Materials and methods

### Subjects characteristics

Sixteen female patients with a histologically confirmed diagnosis of primary breast cancer admitted at the National Cancer Institute (NCI) were recruited. About 10 mL of peripheral blood was sampled before the administration of any chemotherapeutic treatment or surgical intervention. Clinicopathological characteristics of BC patients are shown in Table 1. About 10 mL of peripheral blood were also sampled from ten healthy donors to be used as control samples. These samples were used for experiments involving freshly isolated NK cells.

**Table 1:** Clinicopathological characteristics of the BC patients

Breast Cancer Patients				Age (years)		*Number (%)*		
				≥ 45		10 (62.5%)		
				< 45		6 (37.5%)		
Invasive carcinoma histological grade	I	0 (0%)	Tumor Stage (size of tumour)	T1	2 (12.5%)	Has the tumor spread to the lymph nodes?	N0	(0%)
	II	13 (81.25%)		T2	7 (43.75%)		N1	14 (87.5%)
	III	2 (12.5%)		T3	2 (12.5%)		N2	1 (6.25%)
	Unk	1 (6.25%)		T4	5 (31.25%)		N3	1 (6.25%)
Lymph node metastases	M0	9 (56.25%)	Molecular Subtype	Luminal A			12 (75%)	
	Mx	7 (43.75%)		Non-luminal Her2 +			4 (25%)	
ER status	+ ve	12 (75%)	PR status	+ ve	12 (75%)	Her2/neu status	+ ve	12 (75%)
	-ve	4 (25%)		-ve	4 (25%)		-ve	4 (25%)

*ER* Estrogen Receptor, *PR* Progesterone Receptor, *Her2* Human Epidermal Growth Factor Receptor 2

To obtain sufficient amounts of total RNA needed for m^6^A RNA immunoprecipitation, which is usually 50μg according to the MeRIP kit, equal amounts of total RNA from controls were pooled together. Three control pools were made. The same was applied for BC samples. Three BC pools were made. The 1st pool included 7 patients, the 2nd pool included 4, and the 3rd pool included 5 patients. All pools contained approximately 50μg starting total RNA.

Moreover, about 200 mL of peripheral blood was also drawn from healthy donors for the purpose of establishing primary human NK cell cultures. The study was approved by the local ethical committees of the National Cancer Institute (NCI), Egypt, the German University in Cairo, Egypt, and the Leibniz Research Centre for Working Environment and Human Factor, Germany. Informed written consents were obtained from all volunteers who participated in this study, in accordance with the Declaration of Helsinki. The study is compliant with all relevant ethical regulations regarding research involving human participants.

### Bioinformatics

The mRNA sequences of the NKG2D-signaling pathway genes (NKG2D, DAP10, PIK3, VAV, Rac, Pak, MEK and ERK) and the lytic dependent and independent pathway genes (PRF1, GZMH, FASL, TRAIL) in NK cells were obtained from the National Center for Biotechnology Information (NCBI) Nucleotide database in FASTA format.

These mRNA sequences were analyzed to detect the consensus motif of DRm^6^ACH ([D = A/G/U][R = A/G]*m*^*6*^*A*C[H = U/A/C]) using RNA methylation prediction online tools. First, the Sequence-Based RNA Adenosine Methylation Site Predictor (SRAMP) [24] was used followed by the prediction of N^6^-methyladenosine sites in RNA sequences via Physical–Chemical properties (pRNAm-pc) [39]. The SRAMP tool was accessed at https://www.cuilab.cn/sramp [24] and the pRNAm-PC web server was available at http://www.jci-bioinfo.cn/pRNAm-PC [39] (Access date: June 2018). Both tools predicted mammalian m^6^A sites with high confidence, positioned in the 3’UTR, proximal to the stop codon, in all the mRNA transcripts of interest.

To further identify m^6^A sites, these mRNA transcripts were analyzed using the m^6^A methyl-transcriptome sequencing database (Met-Db V2.0), a database of experimentally detected transcriptome methylation in mammalian primary T cells [39]. The MeT-DB V2.0 web server was accessed at: http://compgenomics.utsa.edu/MeTDB and www.xjtlu.edu.cn/metdb2 (Access date: June 2018).

The results from Met-Db V2.0 were run in a BLASTn search, and their sequences were aligned against the human transcript database to determine the exact m^6^A positions. The predictions from SRAMP and pRNAm-pc tools were compared with the experimental results from MET-DB.

RNA transcripts harboring the highest number of m^6^A sites in the 3’UTR, predicted with high confidence by both RNA prediction tools and Met-Db V2.0, were selected for m^6^A level analysis in human NK cells. The chosen transcripts were NKG2D, PIK3, VAV1, PAK1, ERK2, PRF1, GZMH, FASL and TRAIL.

For each gene of interest, approximately 100–200 nucleotide sequences were selected based on the m^6^A site frequency and their proximity to the 3’-UTR across different gene variants. Taqman® Custom Gene Expression Assays were designed to span the predicted m^6^A methylation-enriched sites for the desired m^6^A-containing amplicons.

### Cells and cell culture

Unless stated otherwise, all media and supplements were obtained from Gibco/Thermo Fisher Scientific. Human primary NK cells were isolated and cultured as previously described [3]. Briefly, peripheral blood mononuclear cells (PBMCs) were obtained via density-gradient centrifugation, and primary NK cells were isolated using a Dynabeads® Untouched™ Human NK Cell Kit following the manufacturer’s instructions. NK cells were assessed by flow cytometry and found to be > 95% pure, CD3 − , CD56 + , and NKp46 + .

NK cells were cultivated in Iscove's Modified Dulbecco's Medium (IMDM) + 10% FCS + 1% Penicillin/Streptomycin and supplemented with irradiated K562-mbIL15-41BBL feeder cells, recombinant IL-2 (200 IU/ml, NIH Cytokine Repository, USA) and 100 ng/ml recombinant human IL-21 (Miltenyi, Germany). The cells were incubated in a humidified incubator at 37 °C and 5% CO_2_. After 7 days, NK cells were restimulated with IL-2 and feeder cells and incubated for an additional 7 days. The NK cells were used after approximately 3 weeks of expansion.

For experiments where cultured primary NK cells were treated with Meclofenamate (MA; Cayman Chemical- Biomol GmbH, MI, USA), NK cells were incubated with MA supplemented with IL-2 and IL-15 for 48 h before any experiment was conducted. To assess protein expression, m^6^A levels or mRNA levels of the target genes, NK cells were incubated with 12.5 μM MA for 48 h (Figure S1& S2 in Supplementary File). Likewise, NK cells were incubated with 0.005% v/v Dimethyl sulfoxide (DMSO) as a solvent control.

### CRISPR/CAS9-mediated knockouts (KOs)

TrueCut Cas9 Protein v2 (120 pmol) was mixed with two TrueGuide synthetic guide RNAs (150 pmol each; ALKBH5: 5’-GACGUCCCGGGACAACUAUA-3’ and 5’-GCGCAAGUAUCAGGAGGACU-3’, FTO: 5’-CCGGUAUCUCGCAUCCUCAU-3’ and 5’- GCUUAUUUCGGGACCUGGUU-3’) and delivered into 4 million expanded primary NK cells via nucleofection by Amaxa® 4D Nucleofector® X Unit Technology (Lonza Walkersville, Inc. Houston TX, USA) using program DK 100. After 7 days, transfection efficiency was assessed by western blotting (Antibodies in Supplementary Table 1).

### N^6^-methyladenosine RNA immunoprecipitation (MeRIP)

N^6^-methyladenosine modifications of individual mRNA transcripts were determined using MeRIP. After immunoprecipitation (IP), recovered m^6^A-enriched RNA was analyzed by gene-specific qPCR assays to assess m^6^A modification levels. Total RNA was extracted from cultured or primary NK cells using TRIzol reagent (Invitrogen, Carlsbad, CA, USA) and purified using the DNA-free™ DNA Removal Kit (Ambion, Thermo Fisher Scientific, MA, USA) following the manufacturer’s instructions.

Briefly, 50μg of the purified total RNA was fragmented using the NEBNext® Magnesium RNA Fragmentation Module (New England Biolabs (NEB), MA, USA), following the manufacturer’s instructions (4 min incubation at 94 °C with fragmentation buffer to shear the RNA into approximately 100-nt fragments). 10% of the RNA was saved as the input control, which included both forms of the fragmented mRNA, the methylated and non-methylated forms.

MeRIP was performed according to the manufacturer’s instructions using the EpiMark® N^6^-methyladenosine Enrichment Kit (NEB, MA, USA) following the manufacturer’s instructions. 15 µg of the fragmented RNA was added to the m^6^A antibody coupled Protein G Magnetic beads and incubated. Subsequently, the beads were washed in different concentrations of salt buffers. After the last wash, RNA was eluted from the beads with RLT Buffer (Qiagen, #79216, Germany).

The m^6^A-enriched RNA was cleaned and concentrated using the Dynabeads MyOne Silane (Life Technologies, #37002D, Germany). The RNA-bound beads were washed twice with 100% ethanol and 70% ethanol, respectively. Finally, the RNA was eluted in 20µL nuclease-free water. The IP fraction contained only mRNA molecules that harbored m^6^A modifications.

To confirm RNA enrichment efficiency, control immunoprecipitation (IP) samples were prepared using two control RNAs provided in the EpiMark N^6^-Methyladenosine Enrichment Kit, one with m^6^A modification (*Gaussia* luciferase) and one without (*Cypridina* luciferase) to monitor enrichment and depletion. 20 nM of each control RNA (m^6^A-modified and unmodified) was mixed and used instead of fragmented RNA. 10 μL of the mix was added to the m^6^A antibody coupled Protein G Magnetic beads for immunoprecipitation, and the leftover mix served as the Input control RNA fraction. 1 μL of RNA from both Input control and IP fractions was reverse-transcribed and analyzed using TaqMan custom assays (Thermo Fisher Scientific) for m^6^A and unmodified controls.

### Reverse transcription—real time PCR (RT-qPCR)

mRNA expression and m^6^A enrichment were analyzed using RT–qPCR. Reverse transcription was performed using the SuperScript™ IV VILO™ Master Mix kit (Invitrogen, Thermo Fisher Scientific, Germany). Quantitative real-time PCR was performed on a CFX96 Real Time PCR Detection System (BIO-RAD, Berkeley, CA, USA) using TaqMan® custom assays for the target transcripts (Life Technologies, Thermo Fisher Scientific, Germany) (Table 2). All qPCR reactions were run in duplicates.

**Table 2:** TaqMan® assays used in qPCR

Assay Type	Assay ID	Assay Name	Cat #
TaqMan™ Gene Expression Assay (FAM)	Hs00420895_gH	*RPLP0*	4331182
Custom Plus TaqMan™ RNA Assay, FAM	ARMFXU3	NKG2D	4441114
Custom Plus TaqMan™ RNA Assay, FAM	ARWCXAM	PI3K	4441114
Custom Plus TaqMan™ RNA Assay, FAM	ARZTEZE	VAV1	4441114
Custom Plus TaqMan™ RNA Assay, FAM	ARRWFJX	PAK1	4441114
Custom Plus TaqMan™ RNA Assay, FAM	ARPRKYY	ERK2	4441114
Custom Plus TaqMan™ RNA Assay, FAM	ARNKTEY	PRF1	4441114
Custom Plus TaqMan™ RNA Assay, FAM	AR2W9KA	GZMH	4441114
Custom Plus TaqMan™ RNA Assay, FAM	ARPRKYW	TRAIL	4441114
Custom Plus TaqMan™ RNA Assay, FAM	ARDJYEH	FASL	4441114
Custom Plus TaqMan™ RNA Assay, FAM	ARU63PR	m^6^A-Control	4441114
Custom Plus TaqMan™ RNA Assay, FAM	ARTZ94U	Unmodified Control	4441114

To measure relative mRNA expression levels, *RPLP0* was used as an internal control (the housekeeping gene) to normalize the data across different samples. The threshold cycle (*C*_T_) of the target gene was normalized to the *C*_T_ of the internal reference *RPLP0* gene using the ‘2^−ΔΔ*C*t^’ method, which provided relative gene expression values [40].

m^6^A enrichment in each immunoprecipitated sample was quantified using the ΔΔ*C*_T_ analysis method, comparing it to non-immunoprecipitated input RNA. All C_T_ values were normalized to an exogenously added m^6^A-modified control (*Gaussia* luciferase), provided in the EpiMark® N^6^-methyladenosine Enrichment Kit (NEB, MA, USA). This RNA m^6^A-modified control was spiked into the samples before RNA fragmentation to monitor m^6^A enrichment.

For every RT-qPCR experiment, No-template controls (NTCs) for each TaqMan assay and No-reverse transcription controls (NRTs) were included to check for contamination and confirm the absence of genomic DNA, respectively.

### Western blotting

Equal numbers of cells were lysed in 1 × Reducing SDS Loading Buffer (Cell Signaling Technology, MA, USA). Proteins were separated by SDS-PAGE using 10% NuPAGE Bis–Tris gels (Thermo Fisher Scientific, MA USA) in MOPS buffer (Invitrogen). After separation, proteins were transferred to a PVDF membrane (Millipore, Germany) and blocked with 5% non-fat dry milk in PBST for 1 h. The membranes were incubated with primary antibodies overnight at 4 °C, washed and incubated with the appropriate HRP-conjugated secondary antibody for at least 1 h at room temperature. The antibodies used are listed in Table S1 in Supplementary File. Western blot images were analyzed using ImageJ software.

### Flow cytometry

To analyze the protein expression changes of the genes of interest, NK cells were stained with Zombie NIR™ Fixable Viability dye (Biolegend, CA, USA). For extracellular protein staining, NK cells were directly stained with fluorescent phycoerythrin (PE) anti-human NKG2D CD314 1^ry^ antibody/PE anti-human CD253 (TRAIL) 1^ry^ antibody (Biolegend, CA, USA). For intracellular staining, NK cells were fixed and permeabilized before staining with FITC-anti-Perforin 1^ry^ mouse antibodies (Biolegend, CA, USA).

Data was acquired on the BD LSR Fortessa ™ Cell analyzer (BD Biosciences, USA) and analyzed using FlowJo software (Treestar). Antibodies used are listed in Table S1 in Supplementary File.

### Functionality assays

#### Degranulation assay (CD107a)

CD107a expression is a sensitive marker for NK cell cytotoxic activity. K562 target cells were co-incubated with NK cells at a 1:1 ratio in the presence of PE-Cy5™ anti-CD107a antibody (BD Biosciences, USA). After 3 h of incubation, NK cells were stained with Zombie Aqua™ Fixable Viability Stain (Biolegend, CA, USA) and NK cell marker stain, Alexa Fluor® 700 anti-CD45 antibody (BD Biosciences, USA). Data was acquired on the BD LSR Fortessa ™ Cell analyzer and analyzed using FlowJo software.

#### Chromium release cytotoxicity assay

Cytotoxicity was measured using a standard 4-h chromium release assay as described before [41], which quantifies end-stage lysis of target cells. Briefly, 5 × 10 ^5^ target cells (T) were labeled in 100μL CTL medium with 100 μCi51Cr (Hartmann Analytic) for 1 h at 37°C, 5% CO_2_. The labeled target cells (5 × 10^3^ cells/well) were added to the NK effector cells in V-bottom 96-well plates. Nucleofected/MA-treated expanded NK effector cells were supplemented with IL-2 and mixed with target cells at different ratios (4:1, 2:1, 1:1 and 0.5:1) in triplicates. After 4 h, supernatants were collected and the released ^51^Cr was measured using a Wizard^2^ (Perkin Elmer) Gamma Counter. Counts from triplicate wells were averaged and then percent specific lysis was calculated using the following equation: % specific lysis = 100 × ((test ^51^Cr release) – (spontaneous ^51^Cr release)) / ((maximal ^51^Cr release) − (spontaneous ^51^Cr release)).

### Statistical analysis

Statistical analyses were performed using GraphPad Prism 6 (GraphPad Software, Inc., San Diego, CA, USA). Experiments were independently repeated at least three times, and all attempts to reproduce the results were successful. For all the bar graphs, data are expressed as mean ± SD. Differences between two groups were analyzed using an unpaired Student’s t-test. *P* values ≤ 0.05 were considered significant (*P < 0.05; **P < 0.001; ***P < 0.0001); *P* values > 0.05; non-significant (NS). To correct for multiple comparisons, we applied False Discovery Rate (FDR) correction using the two-stage linear step-up procedure of Benjamini, Krieger, and Yekutieli, with a significance threshold set at Q = 5% as the standard approach, and Q = 10% only where explicitly stated in the manuscript [42]. FlowJo (Treestar) was used for all flow cytometry data analysis. Sample sizes (biological replicates) and specific statistical tests used are detailed in each figure legend.

## Results

### Bioinformatics results

In reference to the bioinformatics analysis, hypothetical m^6^A sites obtained from SRAMP/pRNAm-pc were validated using the experimental results obtained from the sequencing database MET-Db v2.0. Nine NK-related transcripts—NKG2D, PI3K, VAV1, PAK1, ERK2, PRF1, GZMH, FASL, and TRAIL—were identified as the most methylated as they harbored the highest density of m^6^A consensus motifs.

Next, approximately 100–200 nucleotide sequences were selected for each gene of interest based on m^6^A site frequency and their proximity to the 3’-UTR for the different gene variants. Within these predefined amplicon regions, the following DRACH m^6^A motifs were found: 7 motifs in NKG2D in the 3′UTR, 2 motifs in PI3K, 2 in VAV1, 1 in PAK1, 3 in ERK2, 6 in PRF1, 3 in GZMH, 2 in FASL and 5 in TRAIL. So, the online m^6^A prediction tools and Met-Db confirmed that all nine transcripts were m^6^A modified. These sequences were then used to design customized TaqMan Probe Based Assays for RT-qPCR analysis (Table 3).

**Table 3:**
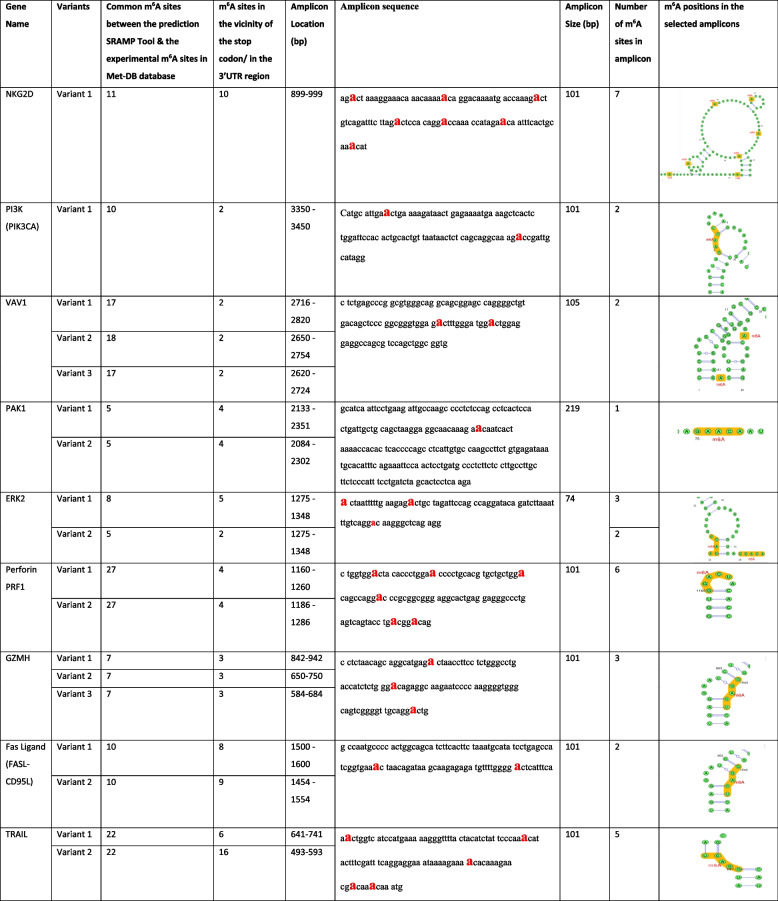
Bioinformatics results. This table presents gene variants, the number of m^6^A sites identified by both prediction tools and the experimental Met-DB database, and the number of m^6^A sites near the stop codon or 3’UTR. It also details the selected amplicon sequence, location, size, predicted number of m^6^A sites within the amplicon, and their positions in 2^ry^ RNA structures. Note: Figures are generated by the bioinformatics tools used, but some m^6^A sites aren’t displayed due to tool limitations shown

### Validation of bioinformatics results

To validate the bioinformatics results, MeRIP-qPCR was performed on expanded 1^ry^ cultured NK cells from three healthy control subjects.

#### Normalization approach

**Fig. 2:**
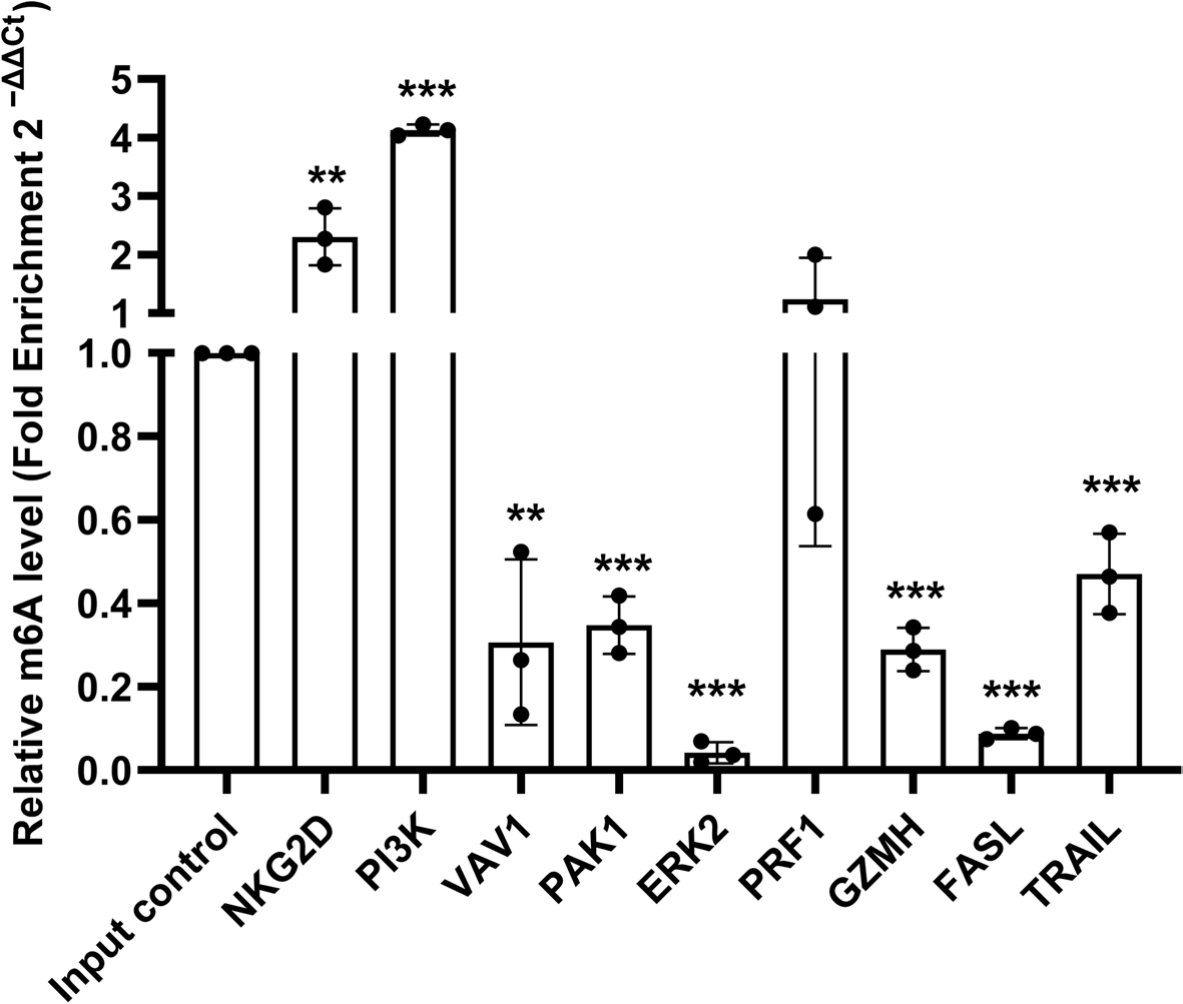
Mean Relative enrichment of target transcripts in 1^ry^ NK cultured cells. To validate the bioinformatics results, MeRIP-qPCR was performed on expanded 1^ry^ cultured NK cells from 3 control subjects (*n* = 3). Relative mRNA m^6^A quantification was conducted by analyzing the m^6^A level in target gene transcripts within the immunoprecipitated (IP) fraction, normalized against the non-immunoprecipitated (non-IPed)/input control RNA. The enrichment in the input control RNA was set to one. C_T_ values were normalized to an exogenously added m^6^A-modified control (*Gaussia* luciferase), provided in the EpiMark® N^6^-methyladenosine Enrichment Kit (NEB, MA, USA). This RNA m^6^A-modified control was spiked into the samples before RNA fragmentation to monitor m^6^A enrichment. Compared to the input control, enrichment in the IP fraction of NKG2D was 2.3 folds higher (*P* = 0.0098, q = 0.0013), and the PI3K was fourfold higher (*P* & q =  < 0.0001), indicating that the m^6^A consensus motifs are significantly enriched in the 3’UTR of NKG2D and PI3K. PRF1 exhibited only a slight increase in m^6^A enrichment within the IP fraction, measuring 1.2-fold higher (*P* = 0.5853, *q* = 0.0683) than the input control fraction, but this difference was not statistically significant. In contrast, several transcripts showed significantly lower m^6^A levels in the IP fraction relative to the input control. VAV1 was 3.3-fold lower (*P* = 0.0037, *q* = 0.0006), PAK1 was 2.9-fold lower (*P* & *q* < 0.0001), ERK2 was 24-fold lower (*P* & *q* < 0.0001), GZMH was 3.5-fold lower (*P* & *q* < 0.0001), FASL was 11-fold lower (*P* & *q* < 0.0001), and TRAIL was twofold lower (*P* = 0.0007, *q* = 0.0001). The significantly lower m^6^A levels in the IP fraction for VAV1, PAK1, TRAIL, ERK2, FASL, and GZMH suggest that these transcripts are minimally methylated in this region. Statistical analysis was performed using a one-sample t-test to determine whether the mean enrichment in the IP fractions was statistically different from the input control, which was set to one

Data in Fig. 2 represent the mean relative enrichment of target genes in IP samples, normalized to non-immunoprecipitated (non-IPed)/input control RNA. The input control RNA enrichment was set to one.

#### Findings

Results in Fig. 2 shows significant m^6^A enrichment in the 3’UTR of NKG2D and PI3K. PRF1 exhibited slight m^6^A enrichment in the IP fraction relative to the input control fraction but was not statistically significant. The m^6^A levels of VAV1, PAK1 and TRAIL in the IP fraction were approximately halved relative to the input fraction, suggesting that around 50% of their transcripts were methylated and 50% were not. ERK2, FASL and GZMH showed nearly no enrichment in the IP fraction, indicating that these transcripts were not methylated in this region. According to a previous study, when fold enrichment is less than or equal to 0.5, transcripts are considered non-methylated [43]. So, based on this criterion, NKG2D and PI3K were found to be significantly methylated, PRF1 was slightly methylated, while the remaining six transcripts were non-methylated.

### Altered m^6^A levels in transcripts of NKG2D, ERK2 and PRF1 in freshly isolated NK cells of BC patients compared to controls

**Fig. 3:**
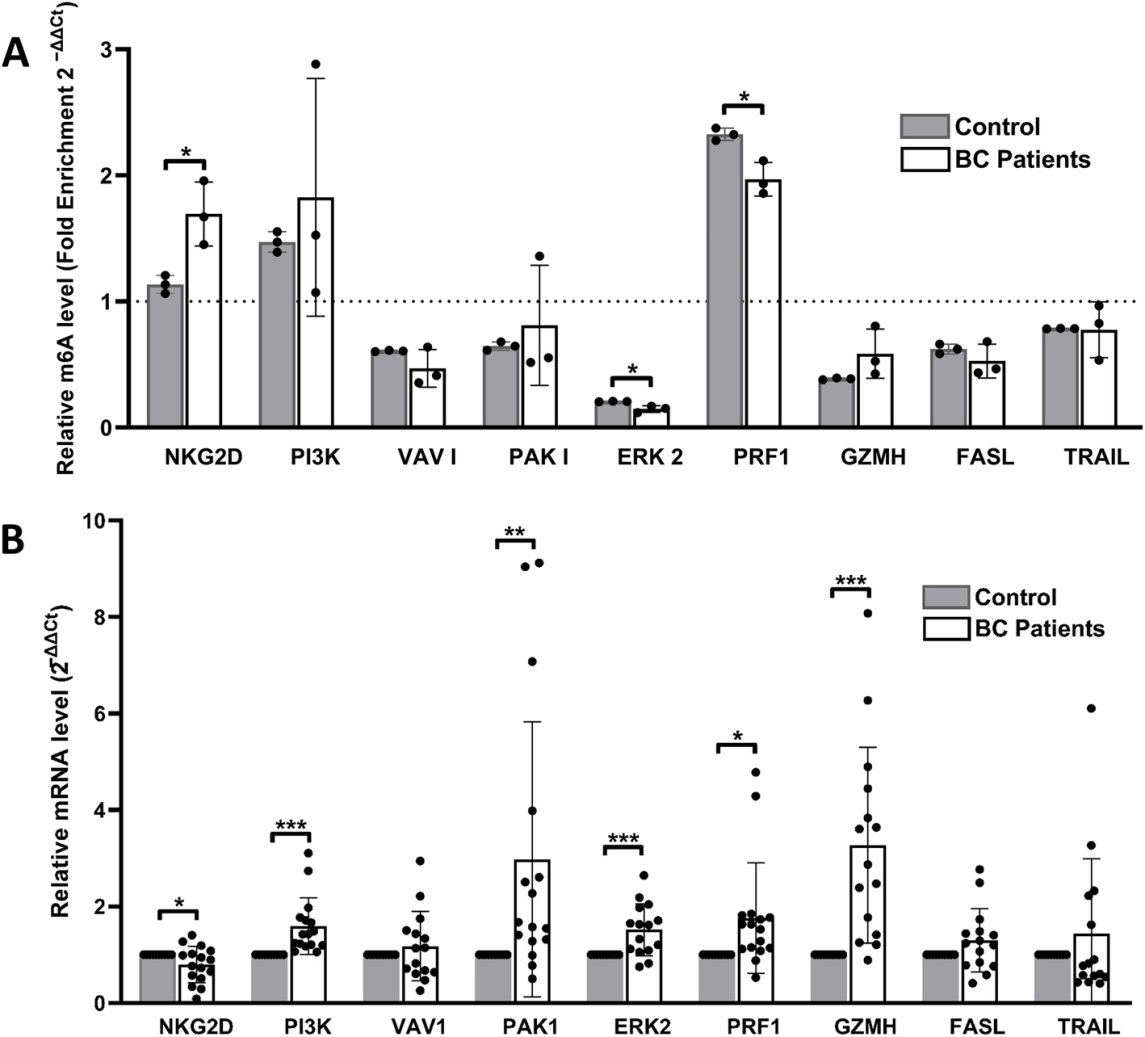
Relative enrichment and mRNA expression of target transcripts in BC patients and controls. **A** MeRIP-RT-qPCR was used to analyze the mRNA enrichment of target genes, comparing the level of m^6^A modification in NK cell transcripts between BC patients and healthy controls. The dotted line represents the non-IP fraction (input control), which is set to 1. C_T_ values were normalized to an exogenously added m^6^A-modified control (*Gaussia *luciferase), provided in the EpiMark® N^6^-methyladenosine Enrichment Kit (NEB, MA, USA). This RNA m^6^A-modified control was spiked into the samples before RNA fragmentation to monitor m^6^A enrichment. Quantitative m^6^A analysis reveals significant alterations in the m^6^A levels of NKG2D, ERK2, and PRF1 transcripts in NK cells from BC patients pools (*n*=3) compared to controls (*n*=3). In BC patients, m^6^A levels were higher in NKG2D mRNA and lower in PRF1 mRNA compared to healthy controls. Although ERK2 m^6^A levels were already very low in controls, they were even lower in BC patients. No significant changes were observed in the m^6^A levels of other target gene transcripts in NK cells between BC patients and controls. Results are expressed as the mean fold change in gene expression ± SD. Differences between groups were analyzed using an unpaired Student’s t-test. **B** RT-PCR was used to assess the mRNA levels of target genes in BC patients (*n*=16) compared to healthy controls (*n*=10). To measure relative mRNA expression levels, RPLP0 was used as an internal control (the housekeeping gene) to normalize the data across different samples. Results are expressed as the mean fold change in gene expression ± SD, and statistical differences were analyzed using an unpaired Student’s t-test

We then wanted to compare the level of m^6^A modification of the NK cell transcripts in BC patients and healthy controls. We isolated NK cells from BC patients (*n* = 16) and healthy controls (*n* = 10), followed by MeRIP-qPCR. Compared to healthy controls, there was an increase in NKG2D m^6^A levels in BC patients, while PRF1 m^6^A levels were lower in BC patients than in controls. Despite the already low m^6^A levels of ERK2 in healthy controls, the levels in BC patients were even further reduced, as illustrated in Fig. 3A. No significant changes were observed in the m^6^A levels of the other target genes. Statistical data for these findings (in Fig. 3A) is presented in Table 4.

**Table 4:** Statistical Interpretation of Data in Fig. 3A

	**Transcript Enrichment in**								
	NKG2D	PI3K	VAV1	PAK1	ERK2	PRF1	GZMH	FASL	TRAIL
Controls IP fraction relative to Input Control	Ns change (*P* = 0.0814)	1.5-fold higher (*P* = 0.0098)	1.6-fold lower (*P* < 0.0001)	1.5-fold lower (*P* = 0.0029)	fivefold lower (*P* < 0.0001)	2.3-fold higher (*P* = 0.0005)	2.6-fold lower (*P* < 0.0001)	1.6-fold lower (*P* = 0.0036)	1.3-fold lower (*P* < 0.0001)
BC Patient IP fraction relative to Input Control	1.7-fold higher (*P* = 0.0417)	Ns Change (*P* = 0.2682)	twofold lower (*P* = 0.0253)	Ns Change (*P* = 0.5620)	sevenfold lower (*P* = 0.0003)	twofold higher (*P* = 0.0062)	Ns Change (*P* = 0.0668)	1.9-fold lower (*P* = 0.0258)	Ns change (*P* = 0.2202)
BC Patient IP fraction relative to Controls IP fraction **	1.5-fold higher (*P* = 0.0212, *q* = 0.0466)	Ns change (*P* = 0.5517, *q* = 0.4809)	Ns change (*P* = 0.1849, *q* = 0.2441)	Ns change (*P* = 0.5829, *q* = 0.4809)	1.4-fold lower (*P* = 0.0173, *q* = 0.0466)	1.2-fold lower (*P* = 0.012, *q* = 0.0466)	Ns change (*P* = 0.1526, *q* = 0.2441)	Ns change (*P* = 0.3018, *q* = 0.332)	Ns change (*P* = 0.9413, *q* = 0.6903)

^**^Q-values (adjusted *p*-values) were obtained using the FDR correction with Q set at 10%

Table 4 provides numerical data and statistical analysis corresponding to Fig. 3A. Each immunoprecipitated RNA fraction, whether from a control sample or a BC patient sample, is always normalized to its respective non-immunoprecipitated (non-IPed)/input control RNA fraction, which is set to 1. Statistics in row 3 (Controls IP fraction relative to Input Control) & row 4 (BC Patient IP fraction relative to Input Control) were obtained by using a one-sample t-test to determine whether the mean enrichment in the IP fractions is statistically different from the input control. Statistics in row 5 (BC Patient IP fraction relative to Controls IP fraction) were determined using an unpaired Student’s t-test to compare BC patients and controls. These statistical differences correspond to the asterisks shown in Fig. 3A.

### Opposing trends in mRNA expression of NKG2D, ERK2, and PRF1

After analyzing m^6^A levels, the mRNA expression of the target genes in BC patients was also examined. As shown in (Fig. 3B), the high m^6^A levels in NKG2D transcripts in BC patients were accompanied by reduced NKG2D mRNA levels (P = 0.0362, q = 0.0253). Conversely, the lower m^6^A levels in ERK2 and PRF1 transcripts in BC patients were accompanied by significantly higher ERK2 (*P* = 0.0005, *q* = 0.0007) and PRF1 (*P* = 0.0126, *q* = 0.0106) mRNA levels in BC patients compared to controls. Additionally, PI3K mRNA levels increased in BC patients (*P* = 0.0003, *q* = 0.0006), and PAK1 (*P* = 0.0093, *q* = 0.0098) and GZMH (*P* = 0.0001, *q* = 0.0004) mRNA expression showed a threefold increase in BC patients compared to controls.

### Altering N^6^-methyladenosine levels by treating 1^ry^ cultured NK cells with meclofenamic acid (MA)

To test the hypothesis that high m^6^A levels reduce mRNA levels, the m^6^A levels were altered in primary cultured NK cells using two approaches. The first involved treating NK cells with Meclofenamic acid (MA), a reported FTO inhibitor [44]. The second employed CRISPR-based Genome Editing to knock out/down the m^6^A erasers, FTO & ALKBH5, individually.

The effect of MA treatment on increasing m^6^A levels in the selected NK cell transcripts was examined, along with its impact on mRNA expression and protein levels. Additionally, the influence of these changes on NK cell cytotoxicity was assessed.

Chromium release cytotoxicity assays and degranulation assays were conducted by incubating cultured NK cells with varying MA concentrations and incubation times to determine the optimal conditions (Figure S1&S2 in the Supplementary File).

#### MA treatment increases m^6^A levels in transcripts of target genes in 1^ry^ cultured NK cells

**Fig. 4:**
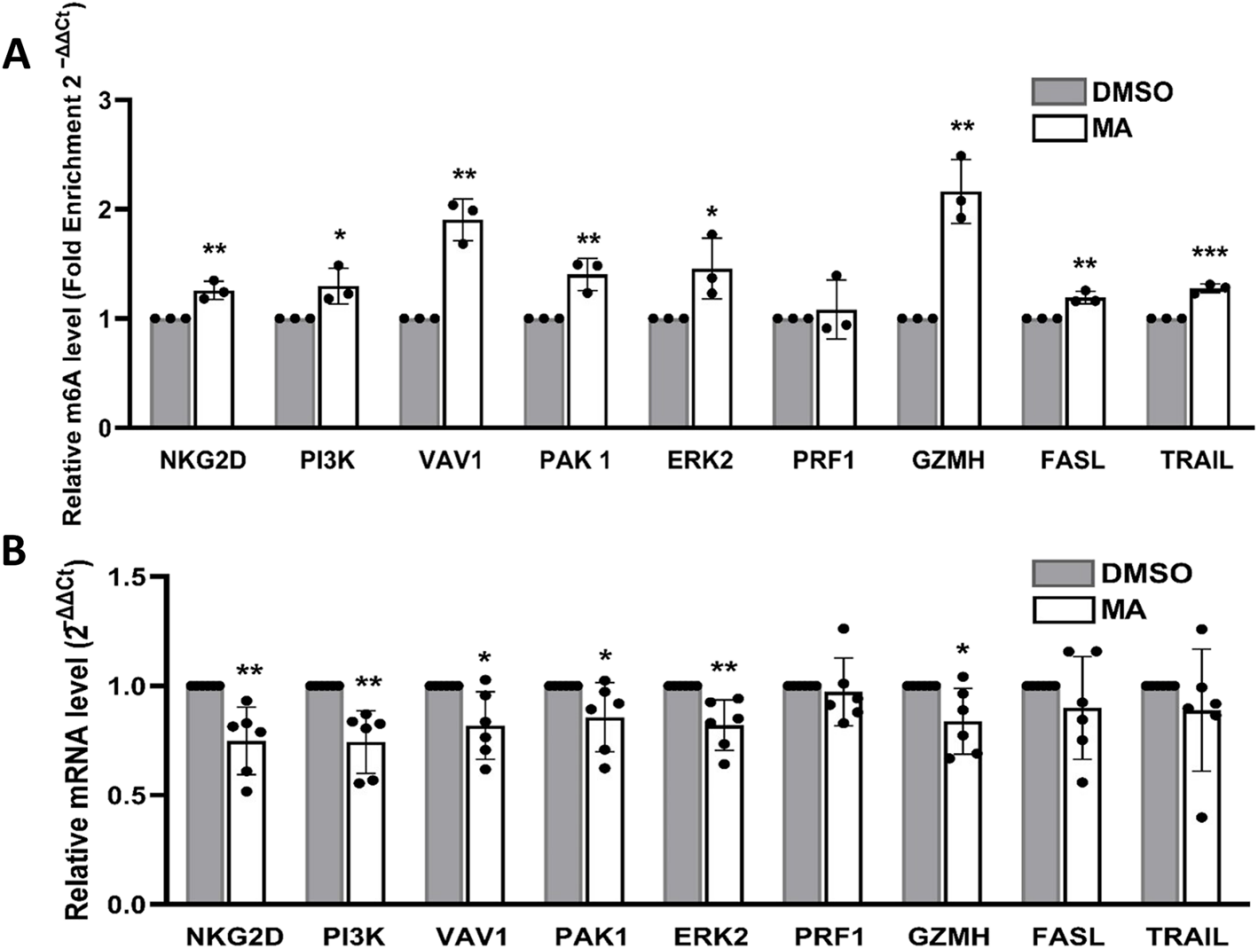
Increasing N^6^-methyladenosine (m^6^A) by MA decreases mRNA expression of target genes in 1^ry^ cultured NK cells. **A** MeRIP-RT-qPCR analysis of mRNA enrichment of the target genes in MA-treated cells compared to DMSO-treated cells. mRNA enrichment in DMSO-treated cells was set to 1. Data is presented as mean ± SD from 3 independent subjects (*n* = 3). C_T_ values were normalized to an exogenously added m^6^A-modified control (*Gaussia* luciferase), provided in the EpiMark® N^6^-methyladenosine Enrichment Kit (NEB, MA, USA). This RNA m^6^A-modified control was spiked into the samples before RNA fragmentation to monitor m^6^A enrichment. **B** RT-qPCR analysis of mRNA expression of target genes in MA-treated cells vs DMSO-treated cells. Data is from six different donors and is presented as mean ± SD (*n* = 6). To measure relative mRNA expression levels, *RPLP0* was used as an internal control (the housekeeping gene) to normalize the data across different samples. Differences were analyzed using an unpaired Student’s t-test to compare the two groups

After incubating NK cells with 12.5 μM MA for 2 days (Figure S1), total RNA was isolated, fragmented, and immunoprecipitated to assess m^6^A levels in the target gene transcripts. An equivalent number of NK cells pre-incubated in DMSO served as controls.

m^6^A enrichment in each immunoprecipitated (IP) sample was quantified using ΔΔ*C*_T_ analysis relative to non-IP input RNA. The DMSO control m^6^A enrichment was set as the baseline (value of one), and the mean relative enrichment levels of target genes were plotted against the DMSO samples (Fig. 4A). As expected, MA treatment (12.5 μM) resulted in a 50%-70% average increase in the m^6^A levels across most target gene transcripts.

#### Deliberate increase in m^6^A levels by MA decreases mRNA expression of target genes in 1^ry^ cultured NK cells

The mRNA levels of most target genes were significantly lower in MA-treated cells compared to those in DMSO-treated control cells (Fig. 4B). This suggests that higher m^6^A levels were accompanied with reduced mRNA expression. These findings align with the trend observed in BC patient data.

#### Deliberate increase in m^6^A levels by MA does not affect protein expression of target genes in 1^ry^ cultured NK cells

**Fig. 5:**
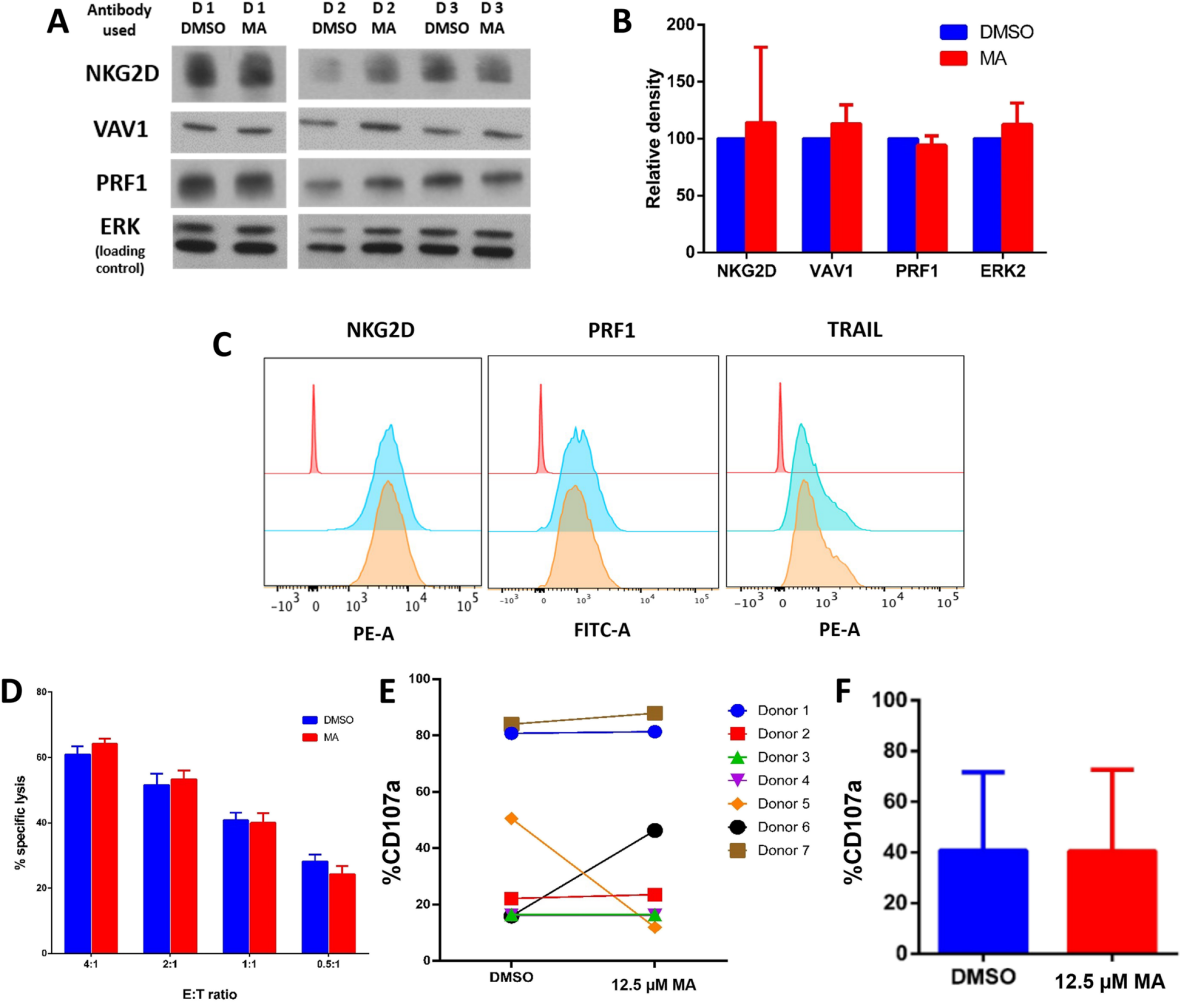
MA-induced m^6^A doesn’t affect the protein expression of target genes or NK cytotoxicity in NK cells. **A** Western blot analysis of protein expression of target genes in NK cells following MA treatment. ERK was used as a loading control. *(Different lanes from the same gel are shown, with cropped sections in the middle. Full-length blots are provided in the Supplementary file).* **B** Quantification of protein expression using relative density by ImageJ. Data is presented as mean ± SD from 3 different donors (*n* = 3). **C** Flow cytometry (FACS) analysis of protein expression in NK cells treated with 12.5 µM MA compared to DMSO-treated cells. Histogram overlays were generated using FlowJo. The red peak represents an unstained control, the blue peaks represent NKG2D, PRF1 and TRAIL protein expression, respectively, in a DMSO-treated sample, and the orange peak represents the same proteins in an MA-treated sample, all from the same donor. Similar results were obtained from 5 different subjects. Controls included a negative unstained control, a positive control for each primary antibody, and compensation controls for each dye (Zombie NIR, NKG2D-PE, PE anti-human CD253, FITC-anti-Perforin). **D** ^51^Cr release cytotoxicity assay showing NK cell cytotoxicity after treatment with 12.5 µM MA compared to DMSO at different E: T ratios. Data is representative of 5 donors and expressed as mean ± SD. Controls: Maximum release (target cells in 1% Triton X-100) as the positive control and spontaneous release (target cells without NK effectors) as the negative control. Percent-specific lysis was calculated based on these controls. **E** Percentage of CD107a expression in 1.^ry^ expanded NK cells from 7 different subjects following DMSO or MA treatment. Controls included a solvent-treated control (DMSO), and NK-only controls (unstained/untreated for auto-fluorescence exclusion and stained/untreated to account for background degranulation). Compensation controls were included for each dye. **F** Mean percentage of CD107a expression in NK cells after treatment with DMSO or MA, expressed as mean ± SD (*n* = 7)

Protein expression levels of NKG2D, VAV1, PRF1 and ERK2 were analyzed using Western blot. No significant differences were observed in the protein expression of any target gene following treatment with 12.5 μM MA from 3 different donors (Fig. 5A and B).

Additionally, NK cells from five donors pre-treated with 12.5 μM MA were assessed for cell surface expression of NKG2D and TRAIL proteins, as well as PRF1 protein levels, using flow cytometry. No significant differences in protein expression were detected between 12.5 µM MA-treated and control NK cells (Fig. 5C). These findings were consistent with the Western blot results.

#### Deliberate increase in m^6^A levels by MA does not affect NK cell cytotoxicity

To determine whether increased m^6^A levels following 12.5 μM MA treatment impacted NK cell cytotoxicity, cytotoxicity assays were performed. No significant differences in percentage-specific lysis by NK cells were observed across four different effector-to-target (E: T) ratios (Fig. 5D).

Furthermore, the percentage of CD107a expression, a marker of NK cell degranulation, was analyzed in NK cells from seven different subjects treated with MA or DMSO. No significant differences were found between the two groups (Fig. 5E&F).

### Altering  N^6^-methyladenosine levels by knocking out (KO) demethylases in 1^ry^ cultured NK cells

**Fig. 6:**
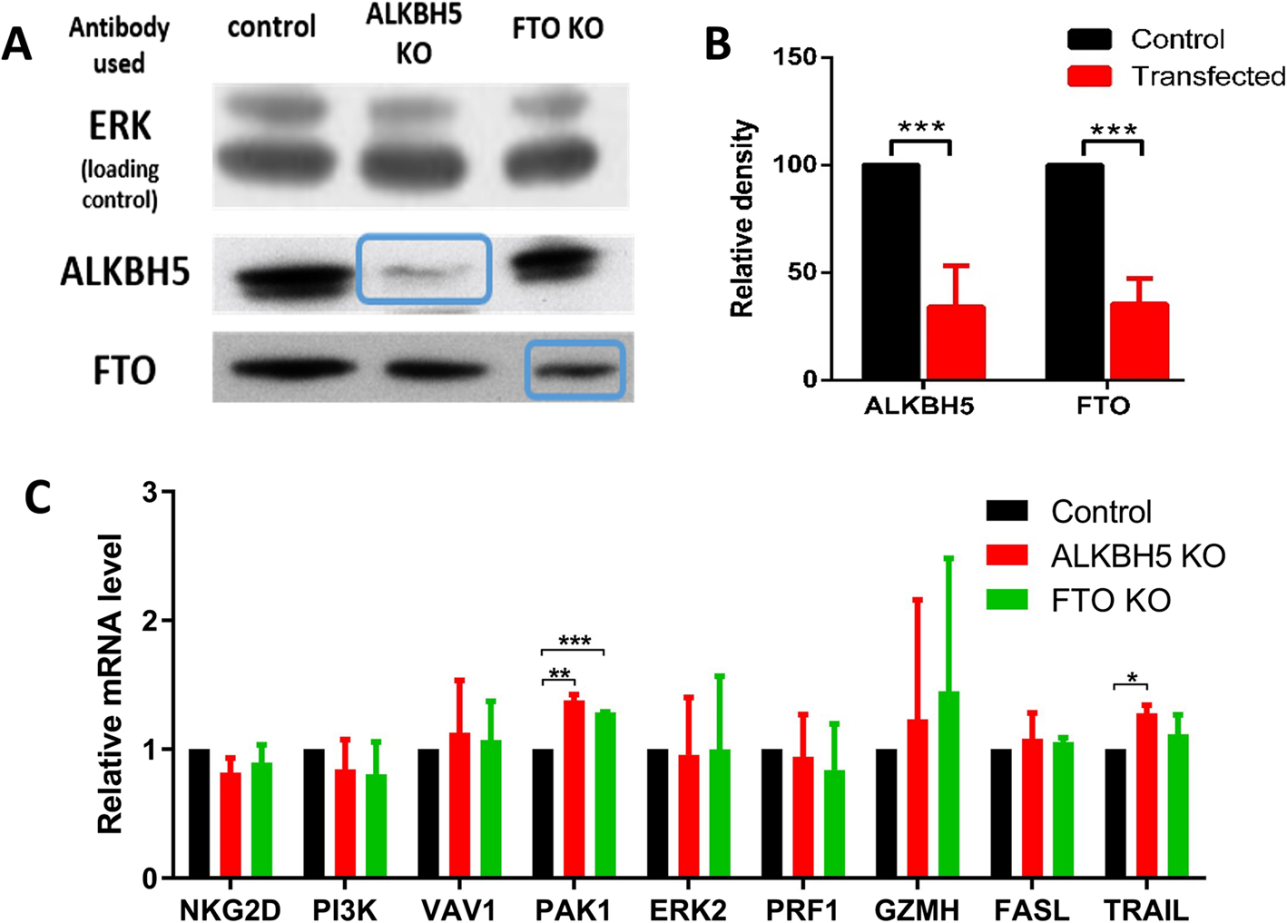
Knockdown of demethylases in 1^ry^ cultured NK cells doesn’t regulate mRNA expression of target genes. **A** Western blot analysis of a demethylase knockout in NK cells from a representative trial. ERK was used as a loading control. *(Different lanes from the same gel are shown. Full-length blots are provided in the Supplementary file).* **B** Quantification of demethylase protein expression using ImageJ after four knockout trials, assessed seven days post-knockout to test knockout efficiency. Data is presented as mean ± SD. **C** RT-qPCR analysis of mRNA levels of target genes in demethylase-knockdown cells compared to control untransfected cells (electroporated without synthetic guide RNAs). Data is presented as mean ± SD (*n* = 2). To measure relative mRNA expression levels, *RPLP0* was used as an internal control (the housekeeping gene) to normalize the data across different samples. Differences were analyzed using an unpaired Student’s t-test to compare the two groups

To investigate the role of m^6^A demethylases, the erasers FTO and ALKBH5 were individually knocked out using CRISPR-based genome editing. The success of these knockouts was validated using Western blotting. Figure 6A presents a representative knockout attempt for both demethylases, while Fig. 6B illustrates the transfection efficiency across four knockout trials, expressed as mean ± SD.

#### Knockout of demethylases in 1^ry^ cultured NK cells does not regulate mRNA expression of target genes

To assess the effect of knocking out NK demethylases, mRNA levels of target genes were analyzed post-KO. Results from two different subjects showed that both knockouts produced similar outcomes.

NKG2D, PI3K, ERK2 and PRF1 transcript levels exhibited a slight decrease following knockout. However, these reductions were not statistically significant (Fig. 6C) when compared to control untransfected cells (electroporated cells without synthetic guide RNAs).

Conversely, VAV1, PAK1, GZMH and TRAIL transcript levels showed an increase in response to the knockout. Despite these variations, none of the target genes displayed a change exceeding 1.5-fold. These findings indicate that knocking out demethylases in 1^ry^ cultured NK cells does not significantly impact mRNA expression of the target genes.

#### Knockout of demethylases in 1^ry^ cultured NK cells does not significantly affect protein expression of target genes

**Fig. 7:**
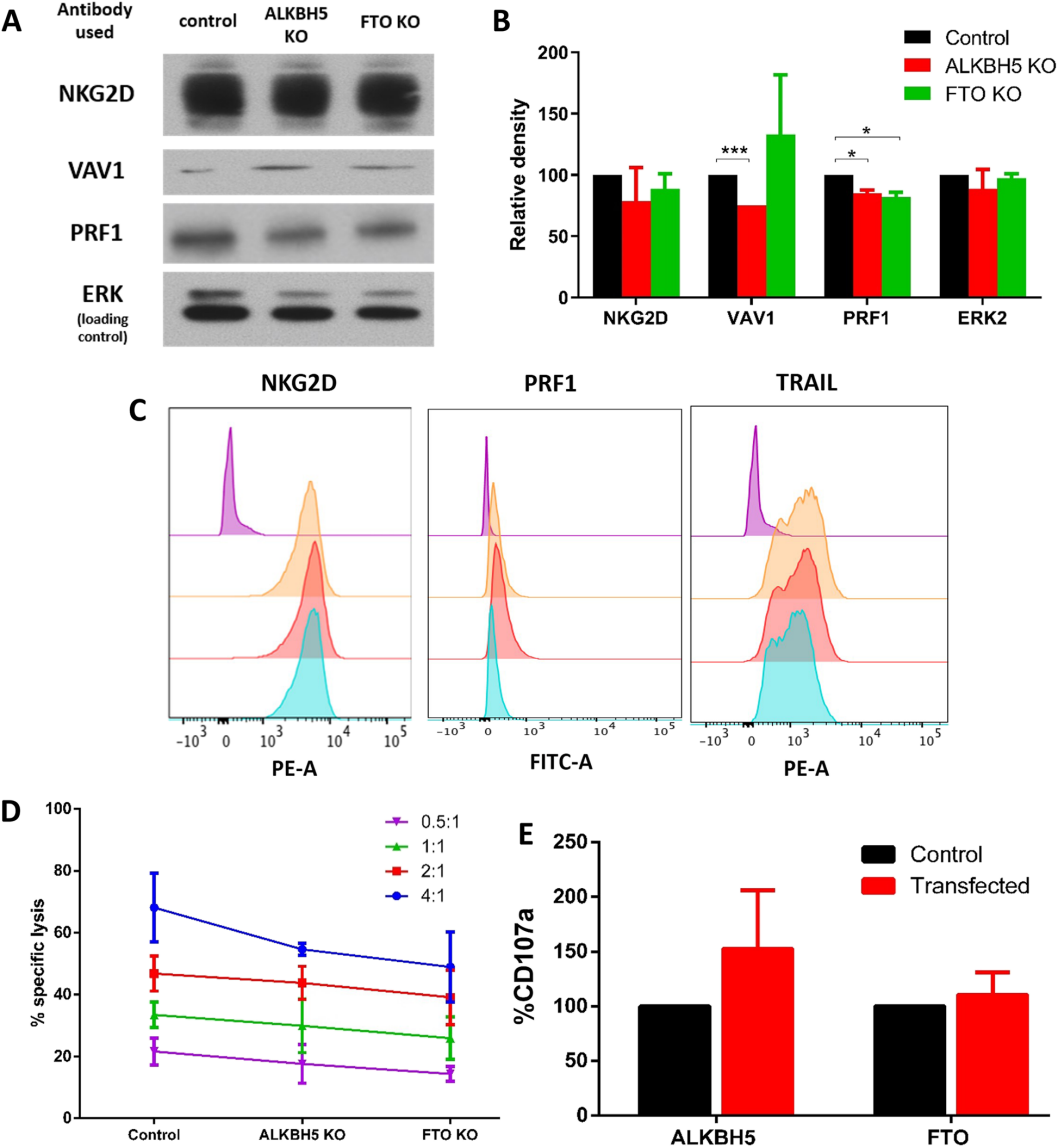
Demethylase knockdown doesn’t affect the protein expression of target genes or NK cytotoxicity. **A** Western blot analysis of protein expression in NK cells after demethylase knockout in one representative subject. ERK was used as a loading control. *(Different lanes from the same gel. Full-length blots are provided in the Supplementary file).* **B** Quantification of protein expression of target genes using ImageJ. Data is from two different knockouts and is presented as mean ± SD. **C** Flow cytometry (FACS) analysis of protein expression in NK cells for NKG2D, PRF1, and TRAIL in transfected NK cells compared to untransfected controls using FlowJo. The purple peak represents an unstained control, the orange peak represents protein expression in an untransfected sample, the red peak represents protein expression in an ALKBH5 knockout sample, and the blue peak represents protein expression in an FTO knockout sample. Data is from a representative subject with similar results obtained in 2 independent KOs. Controls included a negative unstained control, a positive control for each primary antibody, and compensation controls for each dye (Zombie NIR, NKG2D-PE, PE anti-human CD253, FITC-anti-Perforin). **D** Cytotoxicity Assay of demethylases-knock out NK cells against K562 cells, showing mean percentage-specific lysis from 3 donors. Data is presented as mean ± SD (*n* = 3). Controls: Maximum release (target cells in 1% Triton X-100) as the positive control and spontaneous release (target cells without NK effectors) as the negative control. Percent-specific lysis was calculated based on these controls. **E** Mean percentage of CD107a expression in transfected NK cells from 3 different subjects. Data expressed as mean ± SD (*n* = 3). Controls included an untransfected control (electroporated cells without synthetic guide RNAs) and NK-only controls (unstained/untransfected for auto-fluorescence exclusion and stained/untransfected to account for background degranulation). Compensation controls were included for each dye

Protein expression levels of NKG2D and ERK2 remained unchanged between demethylase-KO cells and control untransfected cells (electroporated cells without synthetic guide RNAs). However, VAV1 expression significantly decreased in ALKBH5-KO cells, while PRF1 expression was significantly reduced in both demethylase-KO NK cells (Fig. 7A&B).

Further analysis using flow cytometry measured the expression levels of NKG2D, PRF1, and TRAIL proteins. Results for NKG2D were consistent with Western blot findings. However, unlike the Western blot data, PRF1 expression did not show a significant difference between demethylase-KO and untransfected cells (Fig. 7C).

#### Knockout of demethylases in 1^ry^ cultured NK cells does not significantly affect NK cytotoxicity

To evaluate NK cell functionality, cytotoxicity assays were conducted following ALKBH5 and FTO knockouts using chromium release assays. No significant differences in percentage-specific lysis were observed between control samples (electroporated cells without guide RNAs) and transfected samples (Fig. 7D).

Although a general trend of decreased percentage-specific lysis was noted at a 4:1 effector-to-target (E: T) ratio, the reduction was not statistically significant. Degranulation assay results further confirmed this, showing no significant differences in CD107a expression between control NK cells and ALKBH5 or FTO-transfected NK cells (Fig. 7E).

## Discussion

This project aimed to identify N^6^-methyladenosine (m^6^A) levels in gene transcripts associated with the NKG2D activating receptor signally pathway (NKG2D-PIK3-VAV1-Pak1-ERK2), transcripts of the lytic-dependent pathway (PRF1, GZMH) as well as transcripts in the lytic-independent pathway/ligands of the death receptors (FASL and TRAIL) in NK cells. Additionally, we investigated whether N^6^-methyladenosine levels in these target genes were altered in NK cells of BC patients and how m^6^A influences mRNA and protein expression of these target genes. The goal was to understand the regulatory functions of N^6^-methyladenosine in NK cells to explore its potential in NK cell-based cancer immunotherapy.

### m^6^A levels were altered in transcripts of NKG2D, ERK2 and PRF1 in freshly isolated NK cells of BC patients compared to controls

Compared to healthy controls, there was an increase in NKG2D m^6^A levels in BC patients, while PRF1 m^6^A levels were lower in BC patients than in controls. Despite the already low m^6^A levels of ERK2 in healthy controls, the levels in BC patients were even further reduced, as illustrated in Fig. 3A. No significant changes were observed in the m^6^A levels of the other target genes.

The N^6^-methyladenosine modification serves as a crucial epigenetic regulator of gene expression, playing a pivotal role in immune cell function. While its impact on cancer progression is well-documented [23, 26, 29, 32, 45–59]; its role in immune regulation—particularly in NK cells—has gained increasing attention.

m^6^A modifications influence the innate immune response by modulating inflammatory pathways, cytokine production, and immune cell homeostasis. m^6^A modifications in immune cells, including NK cells, are highly dynamic and can shift in response to various pathological conditions, including cancer, infections, and inflammation. Studies have shown that m^6^A levels fluctuate in response to tumor microenvironments, potentially altering immune cell function [60]. For instance, in some cancers, m^6^A can enhance immune evasion by suppressing antigen presentation in dendritic cells [61] or modulating macrophage polarization toward an immunosuppressive phenotype [62]. Furthermore, viral infections, such as influenza and Rous sarcoma virus, have been found to exploit m^6^A modifications to evade immune detection by suppressing interferon production [61].

These findings collectively suggest that m^6^A modifications are not static but are instead influenced by external triggers, including oncogenic signals, inflammatory cues, and pathogen interactions [60]. Given this complexity, altered m^6^A levels in NK cells of BC patients, as observed in our present study, may reflect broader immunoregulatory mechanisms rather than an isolated phenomenon. Understanding these regulatory dynamics can provide valuable insights into immune modulation in cancer and pave the way for novel therapeutic interventions targeting m^6^A pathways [26, 36].

A key limitation of this study is the use of pooled RNA for MeRIP analysis, which could potentially mask inter-individual variability. However, pooling was necessary due to the high RNA input requirements for MeRIP. To address this concern, we conducted RT-qPCR on both individual BC patient samples and pooled samples for selected genes. Our results demonstrated that mRNA expression trends remained highly consistent between pooled and individual samples, confirming that pooling did not distort biological patterns. Furthermore, equal RNA amounts were used from each patient to minimize bias. While pooled analysis may not capture rare outlier effects, the consistency observed between pooled and individual patient data reassures us that our findings are representative of the broader biological trends. Furthermore, prior studies have reported comparable findings in gene expression profiling when using pooled RNA samples versus individual RNA samples before RNA sequencing or microarray analysis. These studies concluded that pooled RNA samples can effectively identify differentially expressed genes, yielding results similar to those obtained from individual sample analysis [63–67].

### N^6^-methyladenosine influence on mRNA expression of target genes in NK Cells of BC patients

**Fig. 8:**
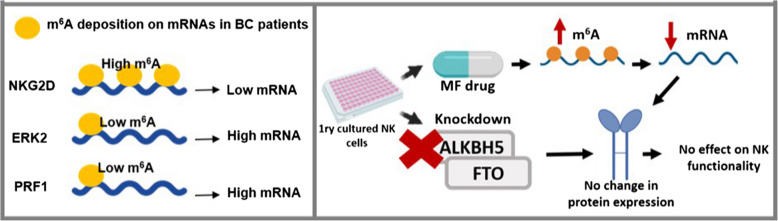
A graphical representation of the results

In BC patients, increased N^6^-methyladenosine levels were accompanied by reduced mRNA levels of target genes, as observed in NKG2D mRNA expression. In contrast, transcripts with lower N^6^-methyladenosine levels, such as ERK2 and PRF1, exhibited increased mRNA levels (Fig. 8). This suggests that the mRNA levels opposed the m^6^A levels, in most cases,

Emerging evidence supports this inverse relationship, particularly when m^6^A is predominantly present in the 3’-UTR [68]. Studies have shown that m^6^A in the 3’UTRs facilitates mRNA degradation by either interfering with the binding of mRNA-stabilizing proteins or by recruiting proteins that target mRNAs to processing bodies (the cellular sites of mRNA decay) [27]. In METTL3 and METTL14 knockdown mouse embryonic stem cells, many METTL3- and METTL14-target mRNAs showed increased stability, suggesting that m^6^A promotes mRNA instability [69]. Another study demonstrated that methylated transcript isoforms have shorter 3′ UTRs and lower stability than non-methylated transcripts, classifying them as “fast-track downstream metabolism” transcripts with accelerated nuclear export, translation, and degradation [70]. This explains why NKG2D transcripts exhibited decreased levels in the presence of higher m^6^A levels.

### mRNA expression of target genes in NK cells in BC patients

The decreased NKG2D mRNA expression observed in BC patients aligns with previous findings by Park et al., where patients with ovarian, breast and cervical cancer exhibited reduced NKG2D expression, leading to impaired NK cell cytotoxicity [16] and tumor progression [18]. Interestingly, NK cells from cancer patients also display an inhibitory phenotype, characterized by upregulated inhibitory markers and downregulated activating markers, including NKG2D and natural cytotoxicity receptors. This inhibitory phenotype is associated with reduced NK cell cytotoxicity compared to NK cells from healthy donors [19].

NKG2D downregulation in cancer patients is mediated by cytokines such as TGFβ and IL‐10, as well as immunosuppressive molecules like prostaglandin E2 (PGE2), vascular endothelial growth factor (VEGF), nitric oxide synthase (NOS), and reactive oxygen species. These factors, produced by tumor cells and immunosuppressive cells (e.g., tumor‐associated macrophages (TAM), regulatory T cells (Treg)), create a chronic inflammatory immunosuppressive microenvironment that inhibits NK cell effector function and facilitates tumor progression.

Another contributing factor to NKG2D downregulation is the persistent stimulation by its ligand-expressing cells or soluble ligands shed by tumor cells. This persistent activation can lead to NK cell exhaustion, impairing NKG2D-mediated cytotoxicity. Consequently, NKG2D appears to be a key target for immune-mediated immunosuppression, and its downregulation is likely a result of tumor immunoediting, which promotes tumor progression [71, 72].

### Impact of lower NKG2D mRNA expression on the NKG2D pathway

The next question is whether reduced NKG2D mRNA expression influences other signaling proteins in the NKG2D pathway. Compared to healthy subjects, BC patients showed significantly increased PI3K, PAK1, ERK2, PRF1 and GZMH transcript levels (Fig. 2).

PI3K mRNA overexpression may be driven by activation through alternative pathways, such as the NKG2C activating receptor, while PAK1 mRNA overexpression may result from activation by the NK Integrin ITGAL/ITGB2 activating receptor. Notably, ERK2 transcripts in BC patients exhibited lower m^6^A levels, which was accompanied by an increased mRNA expression. Since activating receptors other than NKG2D can also activate ERK2, this explains its overexpression [73].

ERK, the final player in the NKG2D-mediated pathway, triggers perforin and granzyme secretion. The observed ERK2 overexpression in BC patients led to an upregulation of PRF1 and GZMH mRNA. These findings are supported by a study showing that after an overnight co-culture with MICA (MHC class I polypeptide–related sequence A)—and ULBP2 (UL16 binding protein 2)-expressing target cells, human NK cells exhibited reduced NKG2D expression and function, but maintained intact cytotoxicity triggered by other activating receptors [74]. Another study demonstrated that expanded NK cells from BC patients retained high cytotoxic activity against BC cell lines and patient-derived tumor cells, similar to NK cells expanded from healthy donors [75].

One key question remains: Are the observed alterations in N^6^-methyladenosine levels in target gene transcripts and their subsequent effects on mRNA and protein expression a consequence of BC, or do they contribute to the disease state?

### Effect of altering N^6^-methyladenosine on mRNA levels in 1^ry^ cultured NK cells

#### Impact of increased m^6^A levels on mRNA stability

Increasing N^6^-methyladenosine levels through either MA treatment or ALKBH5/FTO knockdown produced conflicting results on mRNA expression. The most commonly observed outcome was that higher m^6^A levels led to decreased mRNA levels. This suggests that elevated m^6^A levels on target genes reduce transcript stability, likely inducing transcript decay and ultimately lowering mRNA levels (Fig. 3).

However, when ALKBH5 and FTO were knocked down, PAK1 and TRAIL transcripts showed an unexpected increase in expression (Fig. 5). This deviation from the expected pattern indicates the involvement of an additional regulatory factor, which is discussed shortly.

#### Contrasting effects of m^6^A on mRNA expression

These findings align with earlier studies analyzing differential mRNA expression in METTL3-knockdown cells. Those studies reported that m^6^A stabilizes mRNA, and loss of m^6^A levels correlated with reduced expression of transcripts containing m^6^A [76]. A study using transcriptome RNA sequencing to investigate ALKBH5 knockdown in HTR-8 cells—a first-trimester human extravillous trophoblast-derived cell line—identified 6,891 dysregulated genes. Among them, 3,383 were upregulated, and 3,408 were downregulated, with most showing a fold-change value between 0.5 and 2. This demonstrates that both the overexpression and underexpression of genes can occur following ALKBH5 knockdown [77]. Similarly, METTL3-knockdown studies confirmed that loss of m^6^A correlated with reduced transcript expression, further suggesting that m^6^A plays a role in mRNA stabilization [27].

These findings highlight that mRNA levels are tightly regulated by both transcription and decay mechanisms [24]. While most transcripts are controlled primarily by transcription rate, approximately 17% are significantly influenced by mRNA degradation rates [34]. N^6^-methyladenosine may stabilize certain mRNAs by recruiting reader proteins that enhance transcript stability, preventing degradation and naturally increasing gene expression [76, 78]. This could explain why PAK1 and TRAIL transcript levels increased following FTO and ALKBH5 knockdown. These opposing effects of N^6^-methyladenosine on mRNA regulation indicate its indirect influence on the translation potential of these transcripts [25]. Further research is needed to explore how m^6^A modification affects mRNA fate, particularly in different cell types, to identify the core target genes of m^6^A regulators.

#### Study limitation: challenges in assessing m^6^A levels post-knockout

Another limitation of this study is that N^6^-methyladenosine levels were not assessed following the KO. Measuring m^6^A levels in target gene transcripts post-KO was challenging because the electroporation procedure requires starting with a limited number of cells. Additionally, electroporation significantly reduces cell viability [79], preventing the collection of sufficient total RNA required for m^6^A RNA immunoprecipitation experiments.

Repeating the KO multiple times on cells from the same donor was not a viable solution, as each electroporation experiment introduces various non-specific side effects and results in different transfection efficiencies. Combining cells from multiple electroporation experiments would have created inconsistencies, making it difficult to draw reliable conclusions.

### N^6^-methyladenosine effect on protein expression

#### Lack of correlation between mRNA and protein levels

In this study, changes in mRNA levels did not translate to corresponding changes in protein expression. Protein levels of target genes in MA-treated and demethylase-knockdown cells remained similar to those in control cells. These findings are not surprising, as several studies have consistently shown that cellular protein levels do not necessarily correlate with mRNA levels. Post-transcriptional regulation plays a crucial role in gene expression, often determining protein abundance more than mRNA levels alone [80].

#### Diverse effects of m^6^A on translation

Studies on FTO-knockout mice further support this observation. Five mRNAs with increased m^6^A peaks exhibited slight increases in mRNA levels, but the encoded protein levels showed no consistent trend. In some cases, protein levels decreased, while in others, they remained unchanged or increased slightly [81]. This variability highlights that m^6^A’s impact on translation efficiency remains an unresolved issue.

Research on m^6^A modifications has yielded conflicting findings regarding their role in translation. Many studies suggest that m^6^A enhances translation efficiency [82–84], while others indicate that it may have no effect or even reduce translation [85, 86]. The location of m^6^A modifications within transcripts appears to be a key determinant. When methylation occurs in untranslated regions (UTRs), translation is typically enhanced. However, methylation within coding regions tends to suppress translation [85]. These findings suggest that m^6^A's influence on protein synthesis is not uniform across different mRNAs, and mRNA abundance alone does not dictate translation efficiency [27, 61].

#### The role of reader proteins in m^6^A-mediated regulation

A critical factor in dictating the fate of m^6^A-modified transcripts is the type of reader protein that recognizes and binds to them. m^6^A can potentially exert opposing effects on gene expression, depending on which reader proteins recognize the modification. The orchestrated, coordinated action of these readers ultimately fine-tunes the final protein abundance within cells [24, 25, 87].

#### Regulatory mechanisms beyond mRNA expression

Additionally, research has consistently demonstrated that mRNA expression levels often do not align with protein levels due to various regulatory mechanisms that impact gene expression beyond transcription [88–90]. Protein abundance is influenced by factors such as translation rates, complex post-translational modifications, and protein stability—factors that cannot be directly inferred from mRNA levels alone [91].

A genome-wide study found only weak correlations between mRNA and protein levels, reinforcing that protein expression does not always mirror mRNA levels due to additional regulatory layers [92]. Another study highlighted how independent cellular mechanisms control mRNA and protein levels, emphasizing that while mRNA provides the blueprint for protein production, actual protein abundance is shaped by additional factors such as translation efficiency and protein degradation rates [93]. These regulatory networks vary between cell types and conditions, allowing cells to fine-tune protein synthesis in response to environmental cues and physiological needs.

Collectively, these findings confirm that protein concentration does not linearly follow mRNA concentration. Instead, complex regulatory processes at both transcriptional and post-transcriptional levels dictate final protein expression. This concept aligns with genomic and proteomic research, which consistently shows that mRNA levels alone are insufficient predictors of protein abundance [88].

### N^6^-methyladenosine effect on NK cell functionality

#### No significant change in NK cell function

No significant functional changes were observed in expanded NK cells following MA treatment or transfection (Fig. 8). However, previous research has shown that FTO-deficient NK cells become hyperactivated and exhibit higher cytotoxicity levels. In mice, FTO-KO in NK cells prevented melanoma metastasis in vivo, while in humans, FTO-deficient NK cells enhanced antitumor activity against leukemia [38].

#### Possible explanations for the lack of functional changes

Several factors might explain why NK cell functionality remained unchanged in this study.

Firstly, it was reported that ALKBH5 depletion affects only about 9% of total m^6^A modification sites, suggesting that ALKBH5 targets specific m^6^A modification sites in mRNAs rather than exerting a broad effect [26]. This limited impact may not be sufficient to alter NK cell function. ALKBH5, for instance, is highly expressed in the testes, where it influences spermatogenesis and fertility in mice [31]. However, ALKBH5-deficient mice reach adulthood with only minor reproductive defects, indicating that its demethylase activity is not essential for overall development [94]. In other words, its effect was not major on the phenotype. This suggests that ALKBH5’s effects on cellular function may be minimal in certain contexts.

Another possible explanation is that knocking down one demethylase does not eliminate the activity of the other. The remaining demethylase may compensate for the loss of the other, maintaining mRNA stability and function. This redundancy could explain why knockdown led to only slight changes in mRNA levels and why protein levels and NK cell functionality remained unchanged.

Additionally, the nature of ex vivo-expanded NK cells may contribute to the lack of observable changes. A previous study reported that ex vivo-expanded NK cells exhibit enhanced cytotoxicity against K562 and other tumor cell lines compared to freshly isolated NK cells. This is likely due to the three-week co-culture period with feeder cells [75]. In our study, untreated expanded NK cells already displayed high levels of degranulation and cytotoxicity. Thus, if increasing m^6^A levels—either by MA treatment or demethylase knockdown—were to enhance NK cell functionality, the effect may not be significantly detectable due to the already heightened baseline functionality of the expanded NK cells.

Finally, N^6^-methyladenosine is just one of many post-transcriptional mRNA modifications with regulatory roles that have been discovered or rediscovered in recent years. In addition to m^6^A, other modifications such as m^1^A, m^5^C, pseudouridine and 2′OMe have been identified, and more are likely to be discovered. These chemical modifications decorate different regions of the pre-mRNA (5′ UTR, coding sequence, 3′ UTR, splice sites) and may be regulated individually or combinatorially to influence mRNA fate. This complexity suggests that the effects of MA treatment or demethylase knockdown may have been neutralized by compensatory mechanisms. The interplay between m^6^A and other regulatory pathways could have buffered any potential functional changes, preventing measurable alterations in NK cell activity [24, 70].

#### Study limitation: lack of functional analysis in NK cells from BC patients

Another limitation of this study is that NK cell functionality in BC patients and controls wasn’t analyzed. This was due to the limited volume of blood collected from both healthy donors and BC patients. The limited blood volume was insufficient to isolate a large number of NK cells for functional assays while also obtaining adequate RNA for MeRIP-qPCR analysis.

### Future directions for research

Given the critical role of NK cells in cancer immunosurveillance, examining the differences in m^6^A levels between BC patients and controls is definitely an essential step toward advancing epi-transcriptomic research in NK cells.

Future studies could focus specifically on NK cells from BC patients to better understand the role of m^6^A modifications in their function. One important area of research is exploring the expression of methyl writers and erasers in NK cells from BC patients to determine their potential role in modulating NK cell activity. Expanding NK cells from BC patients and evaluating their cytotoxicity and functionality post-expansion against different breast cancer cell lines could provide further insight into how m^6^A modifications influence NK cell-mediated tumor suppression.

Additionally, modifying m^6^A levels in NK cells from BC patients and reassessing their functionality could help clarify the specific impact of these epi-transcriptomic changes. Understanding how these modifications affect NK cell responses in the context of BC may open new avenues for targeted immunotherapies and improve the effectiveness of NK cell-based cancer treatments.

Achieving stricter m^6^A modifications by knocking out both demethylases simultaneously or targeting multiple writers may prevent compensatory mechanisms that could mask the effects of individual gene modifications. Furthermore, investigating how mRNA chemical modifications interact with various signaling pathways and exploring the interplay between modification regulators and other cellular components will be crucial to understanding their broader implications in both physiological and pathological conditions.

### Clinical applications: m^6^A modifications as biomarkers and therapeutic targets in cancer immunotherapy

m^6^A modifications are key regulators of gene expression, influencing immune cell differentiation, tumor progression, and drug response. In T cells, they control homeostasis and cytokine signaling, with METTL3 depletion impairing homeostatic expansion by stabilizing SOCS genes, which suppress IL-7/STAT5 signaling. These findings suggested that targeted modulation of m^6^A machinery and T cell-targeted delivery of m^6^A-modifying agents could be leveraged to enhance immune cell function in cancer therapies [33, 34]. Given NK cells' functional similarities to T cells, m^6^A modifications may also regulate their activation and persistence in the tumor microenvironment, making them potential targets for enhancing NK-based therapies.

Beyond immune function, m^6^A modifications serve as prognostic biomarkers in multiple cancers [95–97], including lung adenocarcinoma, hepatocellular carcinoma, and acute myeloid leukemia (AML) [96]. Overexpression of METTL3 is linked to tumor progression, reinforcing the relevance of m^6^A in cancer biology. Investigating m^6^A’s role in NK cells could provide novel biomarkers for immune dysfunction, improving patient stratification and treatment optimization strategies [96].

m^6^A modifications also impact drug response, influencing the efficacy of anti-tumor treatments in cancers such as triple negative breast cancer, ovarian cancer, and AML [98]. Recent studies suggest they may serve as biomarkers for radiation response, with genes like *Ncoa4* maintaining elevated m^6^A levels post-exposure. These findings highlight m^6^A’s broader role in cancer treatment optimization, potentially aiding in biodosimetry and therapy personalization [99].

As NK-based therapies, including CAR-NK and cytokine-induced approaches, gain traction in cancer treatment, understanding m^6^A’s influence on NK cells is crucial. Future studies should explore the epitranscriptomic landscape of NK cells in tumors and assess the therapeutic potential of targeting m^6^A-modifying enzymes, such as FTO inhibitors, to enhance NK cell-mediated immunotherapy.

## Conclusions

N^6^-methyladenosine is a research hot spot in life sciences and continues to attract global scientific interest. However, its precise role in NK cells remains largely unexplored. This study found that transcripts with higher m^6^A levels in the 3′ UTR, such as NKG2D, had reduced mRNA abundance, while transcripts with lower m^6^A levels in the same region, such as ERK2 and PRF1, were more abundant. Additionally, cytotoxicity-related transcripts (PI3K, PAK1, and GZMH) were markedly overexpressed in BC patients. However, deliberate alterations in m^6^A levels of target genes did not consistently affect mRNA levels, protein expression, or NK cell functionality. These findings highlight the complexity of m^6^A regulation and suggest that further exploration of its role in NK cell-based cancer immunotherapy is warranted.

## Supplementary Information

Supplementary Material 1.

## Data Availability

No datasets were generated or analysed during the current study.

## Abbreviations

ALKBH5: Alkylated DNA repair protein alkB homologue 5
BC: Breast Cancer
CRISPR: Clustered Regularly Interspaced Short Palindromic Repeats
CT: Threshold cycle
DAP10: DNAX‐activation protein 10
DMSO: Dimethyl sulfoxide
ER: Estrogen Receptor
ERK: Extracellular signal-regulated kinase
ERK2: Mitogen-Activated Protein Kinase 1 (MAPK1)
FACS: Fluorescence-activated cell sorting
FASL: Fas Ligand
FTO: Fat mass and obesity-associated protein
GZMH: Granzyme H
Her2: Human Epidermal Growth Factor Receptor 2
IHC: Immunohistochemistry
IL: Interleukin
IP: Immunoprecipitation
KO: Knockout
m^6^A: N^6^-Methyladenosine
MA: Meclofenamic Acid
MeRIP: Methylated RNA Immunoprecipitation
MEK: MEK: Mitogen-activated protein kinase kinase
METTL14: Methyltransferase-like 14
METTL3: Methyltransferase-like 3
MICA: MHC class I polypeptide–related sequence A
NCBI: National Center for Biotechnology Information
NK: Natural Killer
NKG2D: Natural Killer Group 2 Member D / Killer Cell Lectin Like Receptor K1 (KLRK1)
PAK: P21- activated kinase 1
PI3K: Phosphatidyl-inositol-3-kinase
PR: Progesterone Receptor
PRF1: Perforin
Rac: GTP Binding Protein Rac1
RT: Reverse Transcription
RT-PCR: Reverse Transcription—Polymerase Chain Reaction
SD: Standard Deviation
SRAMP: Sequence-Based RNA Adenosine Methylation Site Predictor
TRAIL: Tumor Necrosis Factor–Related Apoptosis-Inducing Ligand – CD253 (TNF10SF)
ULBP2: UL16 binding protein 2
UTR: Untranslated Regions
