# Supplementary material for: N6-methyladenosine RNA base modification regulates NKG2D-dependent and cytotoxic genes expression in natural killer cells
Source: BMC Med Genomics. 2025 May 19;18:91. doi: 10.1186/s12920-025-02147-y (PMC12090489; doi:10.1186/s12920-025-02147-y)
Supplement: Supplementary file 1 — Supplementary Material 1. [file 12920_2025_2147_MOESM1_ESM.docx]

**Supplementary Data**

**
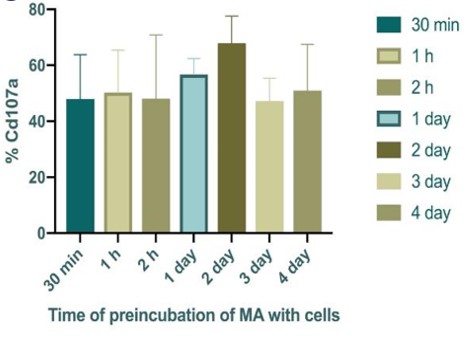
Supplementary Figure 1**

**Figure S1. Kinetics Assay.** Positive CD107a degranulation percentage plotted against different pre-incubation times. Percentage CD107a values are represented as mean ± SD (n=2). Controls included NK-only controls (unstained/untreated for auto-fluorescence exclusion and stained/untreated for auto-fluorescence exclusion). Compensation controls were included for each dye.


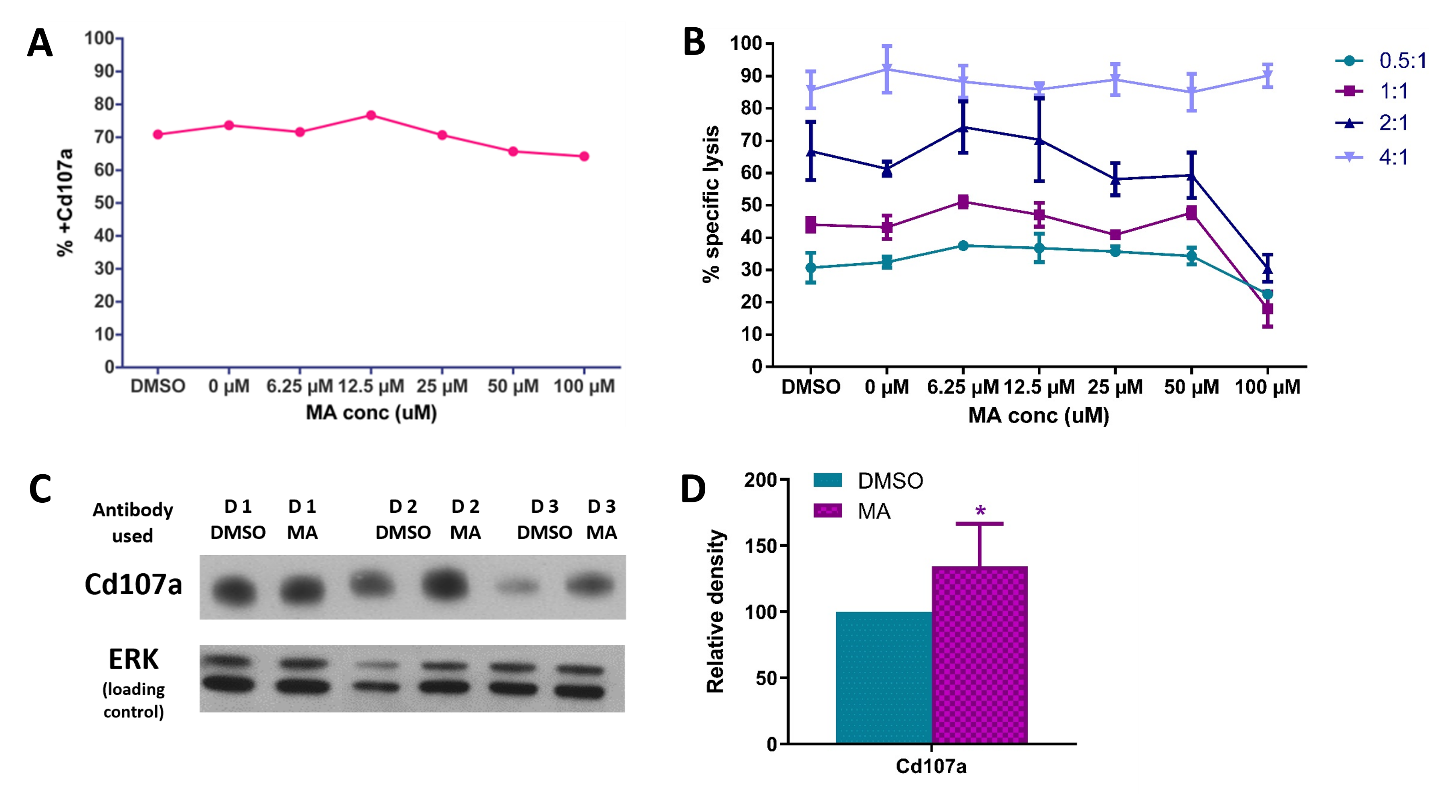
**Supplementary Figure 2**

**Figure S2. A)** Percentage CD107a expression in fresh NK cells plotted against different MA concentrations results from a single representative subject. The highest percentage degranulation is observed at 12.5 μM. Controls included an untreated but stained sample (0 μM MA control), a solvent-treated control (DMSO), and NK-only controls (unstained/untreated for auto-fluorescence exclusion and stained/untreated to account for background degranulation). Compensation controls were included for each dye. **B)** Cytotoxicity Assay of expanded primary NK cells treated with various concentrations of MA against K562 cells. In this figure, a dip in the percentage specific lysis of K562 cells was observed at 25 μM and 50 μM MA at E: T ratio 2:1. Thus, 12.5 μM was the optimum concentration of MA chosen to avoid the potential toxicity that higher concentrations of the MA can cause to NK cells. This is a representative cytotoxicity assay from one donor. Data is presented as mean ± SD. Controls: Maximum release (target cells in 1% Triton X-100) as the positive control and spontaneous release (target cells without NK effectors) as the negative control. Percent-specific lysis was calculated based on these controls. **C)** Western blot analysis showing CD107a protein expression after treating NK cells from 3 different donors with 12.5 μM MA. ERK was used as a loading control. **D)** Protein expression of CD107a of WB in **C** analyzed quantitatively using relative density by ImageJ. Data is presented as mean ± SD from 3 different donors (n=3).

**Table S1.** Sources and applications of antibodies used in this study.

| **Antibody** | **Species** | **Dilution** | **Application** | **Catalogue number** | **Company** |
| --- | --- | --- | --- | --- | --- |
| **1^ry^ Antibodies** | | | | | |
| ALKBH5 antibody [EPR18958] | Rabbit | 1:1000  1:100 | WB  FACS | ab195377 | Abcam |
| FTO Monoclonal Antibody (OTI4A1) | Mouse | 1:1000  1:100 | WB  FACS | MA5-27142 | Thermo Fisher Scientific |
| METTL3 (D2I6O) rabbit mAb | Rabbit | 1:1000  1:100 | WB  FACS | 96391 | Cell Signaling Technology |
| Anti-METTL14 antibody [CL4252] | Mouse | 1:1000  1:100 | WB  FACS | ab220030 | Abcam |
| VAV1 | Mouse | 1:1000 | WB | 05-219 | Millipore/Upstate (Cell signaling solutions) |
| ERK2 (Anti-MAP Kinase 1/2) | Rabbit | 1:1250 | WB | 06-182 | EMD Millipore |
| NKG2D Antibody  Clone 3.1.1.1 | Mouse | 1:1000 | WB | 05-945 | Merck Millipore |
| PE anti-human NKG2D CD314 | Mouse | 1:100 | FACS | 320806 | Biolegend |
| PE anti-human CD253 (TRAIL) antibody | Mouse | 1:100 | FACS | 308206 | Biolegend |
| FITC anti-human Perforin Antibody | Mouse | 1:50 | FACS | 308104 | Biolegend |
| Pacific Blue™ anti-human Granzyme B Antibody | Mouse | 1:100 | FACS | 515408 | Biolegend |
| BD Pharmingen ™ PE-Cy™5 Mouse Anti-Human CD107a | Mouse | 1:200 | WB/ FACS | 555802 | BD Biosciences |
| Alexa Fluor® 700 Mouse Anti-Human CD45  Clone HI30 (RUO) | Mouse | 1:400 | FACS | 560566 | BD Biosciences |
| **2^ry^ Antibodies** | | | | | |
| Goat anti Mouse IgG PE | Mouse | 1:200 | FACS | 115-116-146 | Dianova/ Jackson |
| Donkey anti-rabbit IgG PE (minimal reactivity) Antibody | Donkey | 1:200 | FACS | 406421 |  |
| Horse Anti-mouse IgG HRP linked | Horse | 1:5000 / 1:10000 | WB | 7076S | Cell Signaling Technology |
| Goat Anti-Rabbit IgG HRP linked | Goat | 1:5000 / 1:10000 | WB | 111-035-144 | Jackson ImmunoResearch |
| Goat Anti-Rat IgG HRP linked | Goat | 1:5000 / 1:10000 | WB | 112-036-071 | Jackson ImmunoResearch |

Full-length Blots used for images in the Manuscript.

**For Figure 5A**


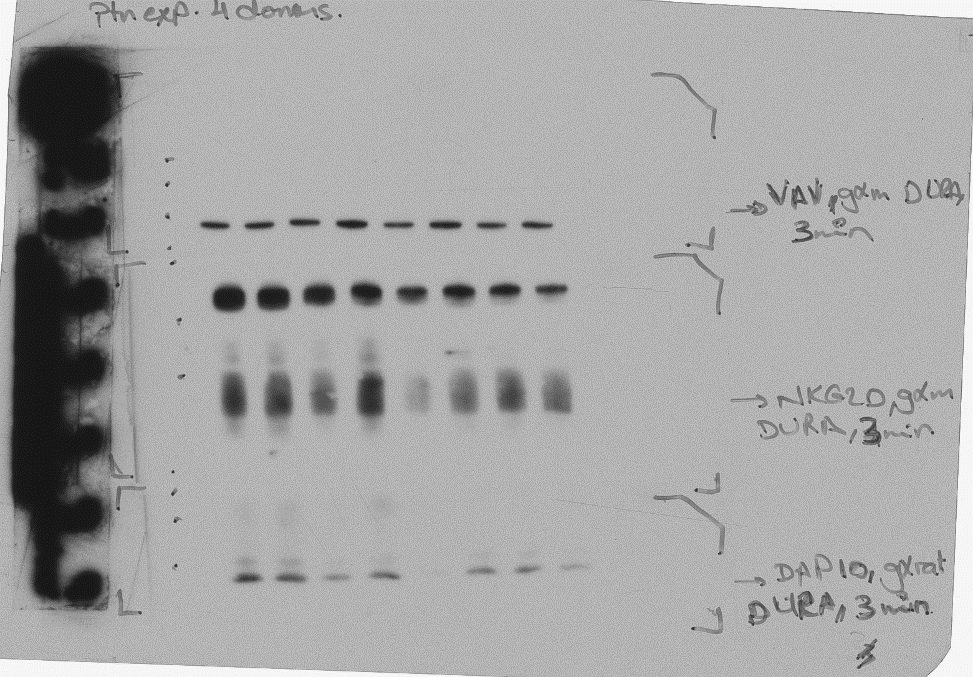


**Figure 1: NKG2D expression in Meclofenamic acid treated NK cells**


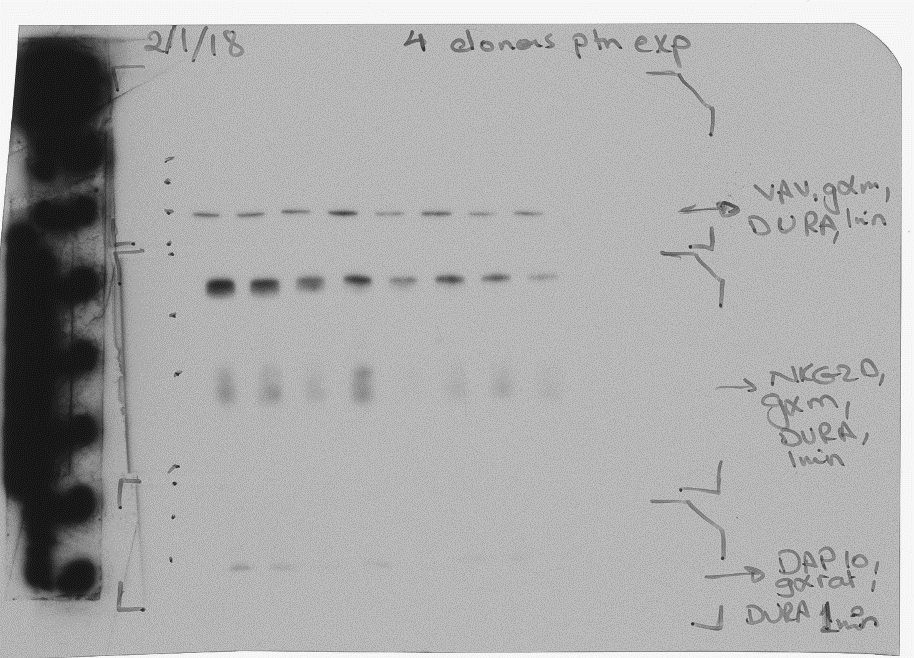


**Figure 2: VAV1 Protein Expression** **in Meclofenamic acid treated NK cells**


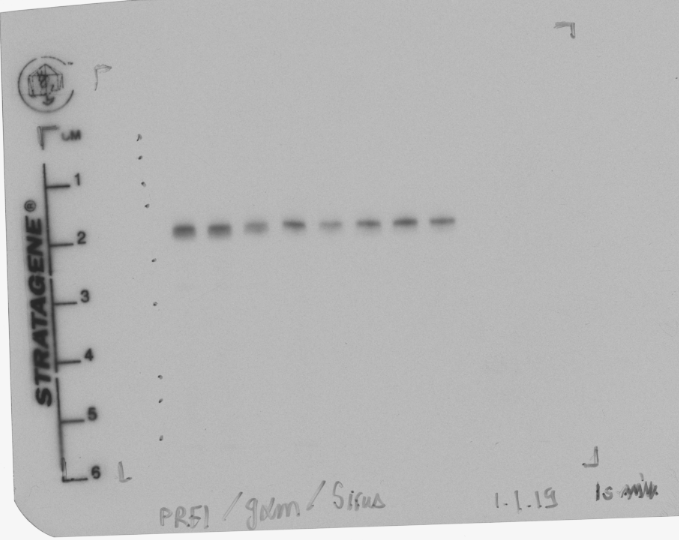


**Figure 3: PRF1 Protein Expression in Meclofenamic acid treated NK cells**


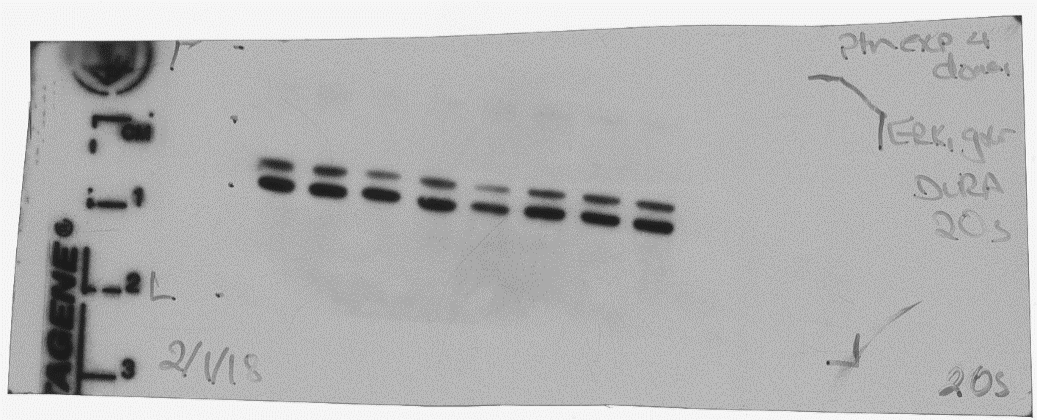


**Figure 4: ERK Protein Expression in Meclofenamic acid treated NK cells**

**For Figure 6A**


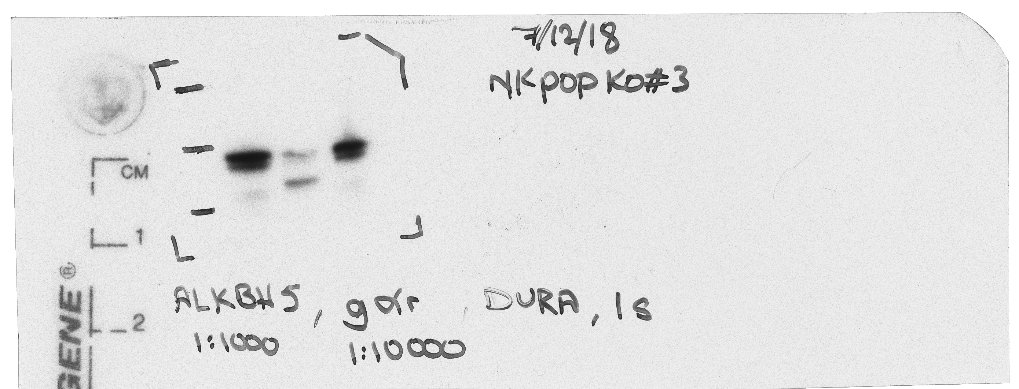


**Figure 5: ALKBH5 Knock Out 2nd lane**


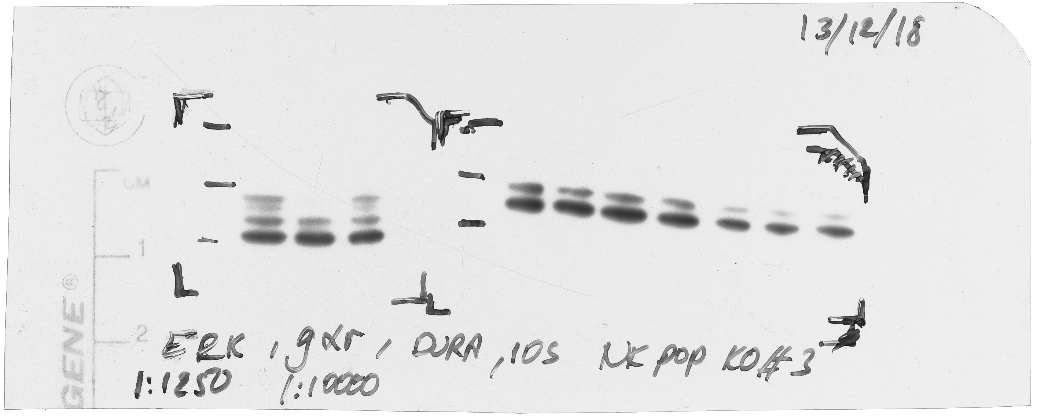


**Figure 6: ERK loading control**


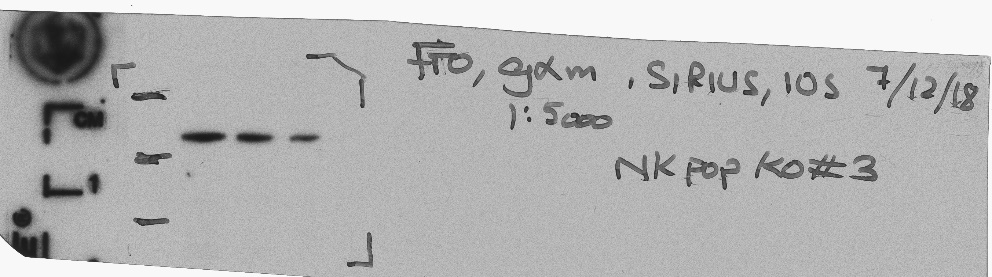


**Figure 7: FTO Knockout 3rd lane**

**For Figure 7A**


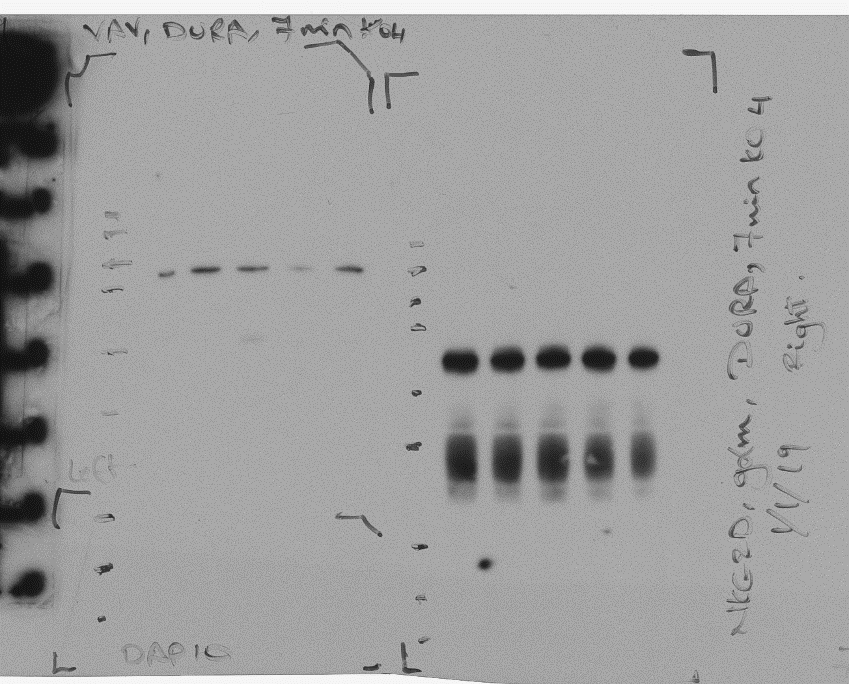


**Figure 8: NKG2D expression (1st 3 bands on the lower right)**


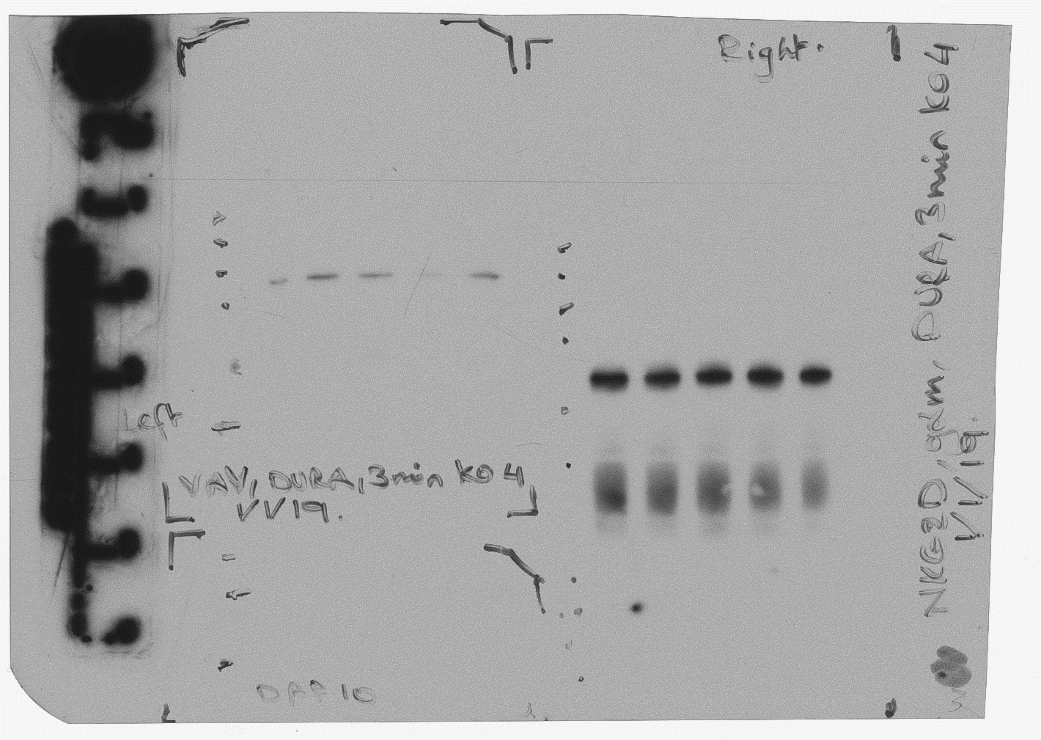


**Figure 9: VAV expression (1st 3 lanes in upper left side)**


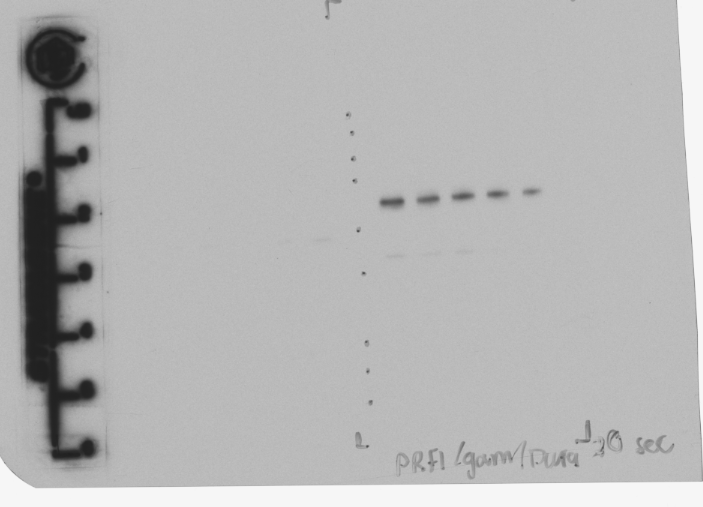


**Figure 10: PFN expression (first 3 bands)**





**Figure 11: ERK expression (first 3 bands on the bottom right quadrant)**
